# Prevention and Health Promotion Interventions for Young People in the Context of Digital Well-Being: Rapid Systematic Review

**DOI:** 10.2196/59968

**Published:** 2024-12-18

**Authors:** Michelle Colder Carras, Dahlia Aljuboori, Jing Shi, Mayank Date, Fatima Karkoub, Karla García Ortiz, Fasika Molla Abreha, Johannes Thrul

**Affiliations:** 1 Department of Mental Health Johns Hopkins Bloomberg School of Public Health Johns Hopkins University Baltimore, MD United States; 2 Carras Colder Carras Ellicott City, MD United States; 3 Health and Social Sciences Singapore Institute of Technology Singapore Singapore; 4 Department of Family and Preventive Medicine Emory University School of Medicine Atlanta, GA United States; 5 Sidney Kimmel Comprehensive Cancer Center Johns Hopkins University Baltimore, MD United States; 6 Centre for Alcohol Policy Research La Trobe University Melbourne Australia

**Keywords:** digital well-being, internet addiction, gaming disorder, social media, screen time, prevention, children, adolescents, mobile phone, PRISMA

## Abstract

**Background:**

Increasing digital technology and media use among young people has raised concerns about problematic use and negative consequences. The formal recognition of a technology addiction (eg, gaming disorder) requires an understanding of the landscape of interventions designed to prevent this disorder and related technology addictions.

**Objective:**

We conducted a rapid systematic review to investigate the current evidence on approaches to prevent problematic technology use and promote digital well-being, defined as the healthy use of digital media and technology and the absence of problems resulting from excessive use.

**Methods:**

We used a pragmatic and rapid approach to systematically review and synthesize recent literature with a focus on contextual factors that can aid in understanding translatability, making trade-offs appropriate for rapid reviews per the Cochrane Collaboration guidelines. We searched multiple databases, including gray literature, for primary studies and systematic reviews of prevention interventions targeting children, adolescents, and youth. We extracted data on study characteristics, quality, and translatability and synthesized evidence through narrative description and vote counting of controlled trials. Data are openly available on our Open Science Framework website.

**Results:**

We found 6416 citations, of which 41 (0.64%) were eligible for inclusion (6 reviews and 35 primary studies of 33 interventions). Most interventions (26/33, 79%) combined intervention approaches and included an education component. Synthesis through vote counting showed benefits for all forms of digital well-being. Both included meta-analyses reported small positive effects on reductions of screen time. However, study reporting was overall lacking, impairing the ability to draw conclusions.

**Conclusions:**

As digital technology use increases, interventions to prevent problematic technology use and promote digital well-being continue to proliferate. Understanding context factors that influence healthy technology use and understanding the limitations of the current evidence are vital for informing future research. This review demonstrates positive findings for the effectiveness of prevention interventions and describes factors that may contribute to translation and implementation. Future research would benefit from following appropriate reporting guidelines, reporting both the benefits and harms of interventions, and including greater detail on factors informing translation.

**Trial Registration:**

PROSPERO CRD42023444387; https://www.crd.york.ac.uk/prospero/display_record.php?RecordID=444387

## Introduction

### Background

The proliferation of digital technologies has ushered new possibilities for connection, communication, and facilitation of life tasks such as work and studying but also concerns about potential issues associated with time spent in front of screens, including excessive gaming and social media use. Problematic use of digital media and technologies is a multifaceted issue encompassing different behaviors and different impacts for various age groups. Although professional organizations have recommendations regarding the use of various types of digital media [1,2], clinically diagnosable problematic digital media use has been characterized as a disorder only regarding video games [3,4]. However, research continues to address other problematic digital behaviors, including addiction to the internet broadly [5,6], social media [7-11], and smartphone use [11-13]. Although there are disagreements about what constitutes normative use and how it differs between media types, media affordances, and generations of users [14], most researchers agree that, for some users, technology- and digital media–related behaviors can cause significant life interference at times, leading to the need for treatment and intervention approaches to promote healthy digital media use.

A growing body of research has started to address this concern through interventions to prevent problematic use and promote digital well-being, which we define as the healthy use of digital media and technology and the absence of problems resulting from excessive use, consistent with other definitions that focus on balancing the benefits and drawbacks to various life domains, such as mental, physical, and social [15,16]. However, the diverse nature of problematic digital media use and the heterogeneity of these interventions make it challenging to evaluate their efficacy [5,17]. While much of the earlier research summarized in previous reviews focuses on excessive involvement in online video games, multiple forms of digital media are now used extensively in daily life, providing a new population norm that differs from that in earlier research on technology addictions.

Previous reviews synthesizing evidence on interventions to prevent problematic use and promote digital well-being face several challenges. Reviewers must contend with a field in which new measures of problematic use continue to proliferate even with 2 very specifically defined technology or media use disorders [17]. In addition, research often takes place outside of disciplines such as public health that have rigid standards for evidence synthesis, leading to incomplete or selective outcome reporting [18]. Context is also a challenge; public and scientific concern about the extent of problematic digital media use varies across global regions, leading to differences in intervention targets and approaches. For example, policy approaches differ between global regions [17,19], and studies about problematic digital media use often downplay contextual factors that may contribute to the feasibility of interventions in various settings [20,21]. Successful public health interventions hinge on a deep understanding of the social and environmental factors that contribute not only to health but also to intervention implementation [22]. As such, understanding context is vital for evaluating what might work to prevent problematic digital media use and promote digital well-being in global settings.

Using frameworks that account for constellations of potential risk factors can provide insights into evidence on prevention interventions [23,24]. To better contextualize potentially modifiable factors that affect the development of digital well-being or problematic use across the life span, we adapted the development of a digital well-being framework [25], which starts from a social ecological model and incorporates cognitive theories [26], media context [27], traditional approaches to the development of addictions (ie, incentive sensitization theory [28]), and uses and gratifications [29].

### Objectives

The aim of this rapid review was to describe the recent literature on interventions used in children, adolescents, and youth (aged <25 years) to prevent problematic digital media and technology use and promote digital well-being, with a focus on understanding the context of interventions and theoretical approaches from a developmental framework. The review methods, including the research question (RQ), search strategy, inclusion and exclusion criteria, and risk-of-bias assessment, were developed a priori and described in the registered protocol (PROSPERO CRD42023444387); these are also available in Multimedia Appendix 1 [8-13,30-58].

## Methods

### Overview

The registered PROSPERO protocol; description of the population, intervention, comparator, outcome, and study design (PICOS) framework; search strategies; and other supporting documentation are available in Multimedia Appendix 1 [8-13,30-58]. The PRISMA (Preferred Reporting Items for Systematic Reviews and Meta-Analyses) checklist is available in Multimedia Appendix 2. Data extraction forms and databases, including a list of articles excluded at the full-text screening stage with reasons for exclusion and all extracted data, extraction of all data, and results of the tool named A Measurement Tool to Assess Systematic Reviews (AMSTAR) checklists, are available on our Open Science Framework (OSF) website [59]. As this study is a systematic review of the literature, it is not considered human participant research.

### Study Design and RQs

Our review included both systematic reviews (henceforth called *reviews*) and primary studies (henceforth called *studies*). As this was not human participant research, institutional ethics approval was not necessary. We used a pragmatic approach to systematically review and synthesize recent literature rapidly (approximately 9 months) with a focus on contextual factors that can aid in understanding translatability, making trade-offs in our methods appropriate for rapid reviews as per recommendations from the Cochrane Rapid Reviews Methods Group [60]. For example, we limited the number of outcomes to two (first, digital wellbeing or problematic use, and second, time spent on digital media); we restricted the search dates; and we used a single-extractor approach to data extraction, with 10% checked by a second team member. We adapted a conceptual model that combines theoretical frameworks focusing on individual development of addictions and media effects as well as models of behavior that combine individual-level and contextual factors [25-29,61].

We addressed the following RQs developed with stakeholder input:

1. What health promotion and prevention interventions have been used to promote digital well-being and prevent problematic digital media and technology use in children, adolescents, and youth?
2. What theoretical or treatment models and approaches inform the development of these interventions?
3. How effective are these interventions when compared with other interventions or no intervention?
4. What characteristics of intervention setting or delivery may limit or promote translation to other contexts?
5. What quality issues should be addressed in future empirical studies of these interventions?

### Search Strategy and Screening

We conducted searches in March 2023 combining terms as described in this section. Citations were downloaded into Zotero (Corporation for Digital Scholarship) [62] and then uploaded into Covidence (Veritas Health Innovation) [63] for screening and inclusion. To update previous literature reviews and allow for a rapid review focus on methodological rigor in data extraction, synthesis, and risk-of-bias assessment, we searched for empirical studies and systematic reviews published since 2017 in all languages in PsycINFO, Web of Science, and PubMed, as well as the gray literature databases of the World Health Organization (IRIS database) and ClinicalTrials.gov. We also hand searched the reference lists of review articles. We piloted combinations of various search terms that included subject and text keywords to retrieve papers related to the following: (1) children, adolescents, and young adults (aged ≤25 years) who did not meet the criteria for technology use disorders (participants); (2) interventions related to promoting healthy digital media use or preventing excessive or problematic use (interventions or exposures); (3) any comparator or no comparator (comparators); (4) digital well-being or the prevention of problematic or disordered use, including symptoms of problematic use and time spent on digital media (outcomes); and (5) primary studies and reviews of interventions (study type).

The final search terms can be found in Multimedia Appendix 1 [8-13,30-58]. We included intervention studies of various designs, protocols, and systematic reviews from all settings that (1) addressed primary or secondary prevention (ie, used a sample that was not formally diagnosed with digital media use disorders, such as internet gaming disorder, where the aim was prevention rather than treatment) *and* (2) contained outcomes related to time using digital media or technology or problematic or excessive use even if these were not primary outcomes. We excluded studies that (1) focused on clinical treatments or a sample with disordered levels of behavior or symptoms (eg, focused on “addicted” individuals), (2) focused primarily on other behavioral addictions, (3) lacked outcomes measuring digital media or technology use or addiction symptoms, or (4) had a mean sample age of >25 years. Titles and abstracts and then full texts were screened independently by 2 raters; discrepancies were resolved through discussion. In cases where study samples seemed to overlap, we contacted the study authors to ensure that the studies were distinct.

### Data Extraction

We developed and pilot-tested a data extraction form based on the standards for rapid reviews [64] and evaluation of complex interventions [65], AMSTAR 2 criteria [66], and the Cochrane Eyes and Vision work group [67]. Data extracted from primary studies included PICOS criteria; theoretical approach, study aims, and design; facilitators or barriers for the study context; possible sources of bias; harms reported; authors’ conclusions; and evaluation of quality and translatability using adapted forms of the Critical Appraisal Skills Programme (CASP) checklists [68,69]. One investigator extracted data from each study, and then a second investigator reviewed the data extraction for all studies, including reviews.

We classified the outcome as the broadest category when categories were combined; for example, “excessive media use” (“exzessive Mediennutzung” [8]) and “GD [gaming disorder] and unspecified IUD [internet use disorder]” [30] were designated as problematic internet use. Where social networking–related categories were combined with another category but were the subject of the intervention, we designated social networking as the focus, for example, “Internet addiction *to the social networks*” (emphasis added) [31].

Efficacy outcome data were extracted from tables, text, and figures in Microsoft Excel (Microsoft Corp) and Stata (StataCorp) to create counts for vote counting for controlled trials only. Where possible, use of digital media was converted to minutes per day (eg, from hours per day) to facilitate comparisons. For studies reporting univariate and multivariate outcomes, we extracted all outcomes.

Data extracted from reviews included citation information, type of review, aims and objectives, target population and setting, eligibility criteria for inclusion and exclusion, methods for specification of outcomes chosen for inclusion, measurement of outcomes, results, authors’ conclusions, and quality and risk-of-bias criteria. The AMSTAR 2 checklist was completed for each review and can be found on our OSF website along with the review data extraction database. Due to time constraints, missing data were not requested from the authors.

### Quality and Reliability Assessment

#### Primary Studies

Because this was a rapid review focusing on context and translatability, we used critical appraisal rather than formal risk-of-bias assessment and scoring to evaluate. CASP checklists [68,69] were adapted for controlled trials, before-and-after studies, and observational studies to focus on addressing quality and translatability outside the original study context. Data were extracted by 2 reviewers independently, and discrepancies were resolved through discussion.

#### Reviews

We adapted an existing approach to quality and risk-of-bias assessment based on a definition of reliability developed by Cochrane Eyes and Vision [67]. This definition specifies that reviews can be defined as *reliable* when they define eligibility criteria for study inclusion; conduct a comprehensive literature search, including articles not in English and multiple search methods; assess the risk of bias of individual studies; use appropriate methods for meta-analysis where conducted; and present conclusions that are supported by the evidence reported in the review. To address potential selective outcome reporting, we added the specification that review authors should have clearly specified in the methods or protocol which outcomes from their eligible studies were included. Reviews were classified as reliable only when all the aforementioned 6 criteria were met. We also conducted a full assessment of review quality using the AMSTAR 2 checklist [66].

### Data Synthesis

Consistent with Cochrane recommendations for a rapid review approach [60], we synthesized evidence through multiple tables and figures and provided a narrative synthesis of findings structured around intervention characteristics and approach, level of prevention, and population and setting. Due to the rapid nature of this review, we conducted vote counting to aggregate results from randomized controlled trials (RCTs) and other controlled studies that directly compared an intervention to no intervention. We compared the number of outcomes favoring the intervention versus control groups (ie, beneficial vs harmful effects), focusing on the direction of the effect rather than on statistical significance per revised Cochrane guidelines (a departure from our registered protocol) [70]. We then conducted a binomial proportion test using the *bitesti* command in Stata to determine the probability of observing the distribution of beneficial versus harmful effects by chance. We used the *cii proportions* command in Stata to estimate the SE and Jeffrey CI for this proportion.

## Results

**Figure 1:**
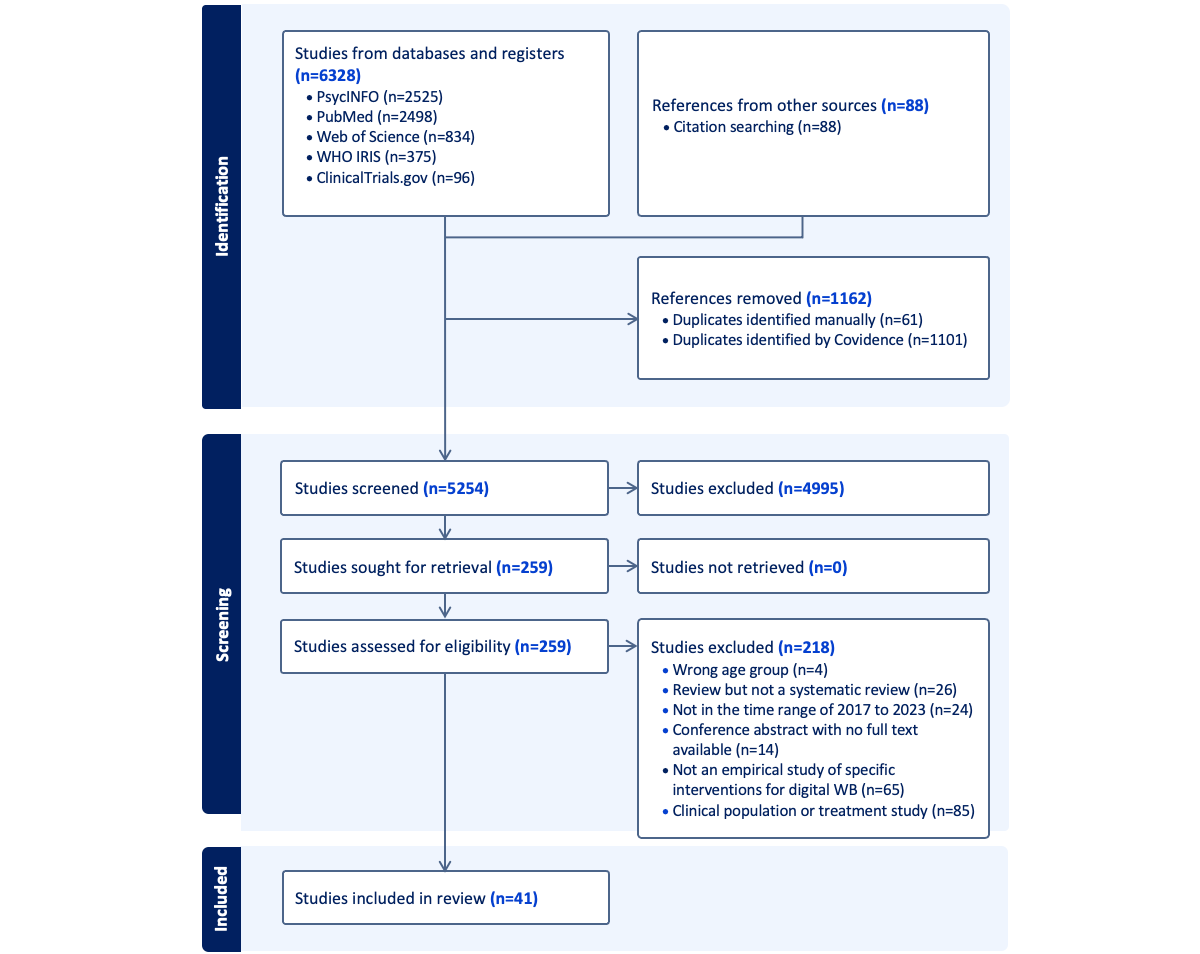
PRISMA (Preferred Reporting Items for Systematic Reviews and Meta-Analyses) flowchart. WB: well-being; WHO: World Health Organization.

The search resulted in 6416 citations, of which 41 (0.64%) were eligible for inclusion (n=35, 85% primary studies and n=6, 15% reviews; Figure 1). The time from completing the search to analyzing and writing up the manuscript was 6.5 months.

### Participants

The studies took place in 19 countries and included between 9 and 243,957 participants, with an average of 8282 (SD 43,744). One national study in South Korea accounted for 243,957 of the participants [32]; when that study was excluded, the maximum number of participants was 2997, and the average was 426 (SD 613). The systematic reviews included between 11 and 204 studies (mean 58, SD 73); 33% (2/6) of the systematic reviews conducted meta-analyses. The systematic reviews included a range of countries, and the total numbers of participants in the systematic reviews ranged from 5627 (calculated) to 162,494 (calculated). The studies focused on children aged 0 to 12 years (3/35, 9% studies), adolescents aged 13 to 18 years (8/35, 23%), and youth aged 19 to 25 years (5/35, 14%), whereas an additional 31% (11/35) of the studies combined more than one age group, 17% (6/35) focused on parents or children and parents, and 6% (2/35) focused on other groups [8,13].

### Study Designs and Comparators

Table 1 provides an abbreviated overview of the characteristics of the studies of individual interventions included in our review; a full summary-of-findings table can be found in Multimedia Appendix 1 [8-13,30-58]. Overall, we found 33 different interventions in 35 studies. A total of 54% (19/35) of the studies were controlled trials that compared interventions to a control group (waitlist or no intervention), 20% (7/35) were before-and-after studies, 23% (8/35) were observational studies or protocols, and 3% (1/35) were unclear [31]. Of the controlled trials, some (3/19, 16%) did not compare extracted outcomes directly between intervention and control groups using statistical tests (eg, compared only within-group changes) and were not included in the vote counting. Of the 27 studies (77%) using causal language in their aims (eg, aiming to “analyze or examine effectiveness or investigate or examine [the] effects” of interventions), several (12/27, 44%) did not use a before-and-after design that compared an intervention group and a control group directly. Most reviews (4/6, 67%) required prospective study designs, but 33% (2/6) included protocols [5,71]; no review included only RCTs.

**Table 1:** Overview of the characteristics of the included studies.

Study					Population and study design		Intervention description		Relevant outcomes
**Controlled studies**									
	**Primary prevention**								
		**Education**							
			Apisitwasana et al [33], 2018	Cluster RCT^a^ of 310 fourth- and fifth-grade students in 2 schools in Bangkok, Thailand		Classroom activities and educational material provided to parents		Game addiction	
			Bickham et al [34], 2018	Controlled before-and-after study of 412 sixth- to eighth-grade students in the United States		Take the Challenge—a school-based media education and use education program		Television viewing, video gaming, and internet use	
			Bonnaire et al [35], 2019	Controlled before-and-after study of 384 middle school students in Paris, France		Single-session prevention intervention designed to increase knowledge and skills		Proportion of gamers with IGD^b^ and minutes per week on the internet and video games	
			Esmaeili Rad and Ahmadi [36]^c^, 2018	RCT comparing intervention to control (no intervention) conducted with 200 college students in Urmia, Iran		Use of a mobile app to decrease social networking addiction		Online social networking addiction and time spent on social networks	
			Gui et al [13], 2023	Cluster-randomized trial of the impact of a teacher training course on 2997 tenth-grade students in 171 classes in 18 schools in northern Italy		DWB-S^d^, a hybrid web-based and in-person training intervention for teachers		Smartphone addiction and social network use	
			Hansen et al [8], 2022	Postintervention survey of 834 students in participating schools in 3 federal states in Germany		The “Net-Piloten” (Net Pilots) peer project for the prevention of social media addiction		Video game play duration, social media chatting time, streaming time, and problematic internet use	
			Li et al [37], 2019	Cluster RCT comparing intervention schools (n=35) to control schools (n=35); 362 parents of fourth- to sixth-grade gamers in Hong Kong, China		GOI^e^, a single-session, 4-hour intervention for parents		Gaming time and gaming disorder symptoms	
			Marco and Choliz [38]^c^, 2017	RCT of 1110 primary and secondary school students in 9 public and 4 private schools in Valencia, Spain		PrevTec 3.1 for video games		Frequency and duration of video gaming and video game dependence	
			Mumcu et al [39]^c^, 2021	RCT of 110 schoolchildren in Turkey		Recreational exercises in school		Game addiction	
			Ortega-Barón et al [40], 2021	Controlled pretest-posttest study of 165 middle schoolers in 3 regions of Spain		The Safety.net program		Problematic internet use	
			Weaver [41], 2022	Pretest-posttest study of 65 students in a single high school in the United States		Mindful Connections		Problematic social media use	
		**Community**							
			Sanders et al [42], 2018	RCT of parents of children between the ages of 5 and 12 years		Single-session, in-person intervention delivered by a therapist to address media parenting		Screen time	
		**Online**							
			Throuvala et al [11], 2020	RCT of 143 university students in the United Kingdom who used mobile phones and social media		An intervention designed to promote mindfulness, raise awareness of media and smartphone use, enhance self-regulation, and reduce distractions and time spent on smartphones and social media		Hours per day of smartphone use, problematic social media use, and hours per day of social media use	
	**Secondary prevention**								
		**Education**							
			Affouneh et al [43], 2021	RCT with 30 university students in West Bank, Palestine, who “engaged in excessive Internet usage”		Group training (CBT^f^-based) to improve social skills and reduce internet-addictive behaviors		Internet addiction	
			Lindenberg et al [30], 2022	Multicenter cluster RCT of 422 high school students in Germany		PROTECT^g^ preventive group intervention		Combined gaming disorder and unspecified internet use disorder (measured using a single modified scale) and intervention harms (number of participants who developed combined gaming disorder and unspecified internet use disorder)	
			Tang et al [44], 2021	Cluster RCT of 775 students at 2 middle schools in Zizhong County, a rural area of Sichuan Province, China		The intervention was primarily provided by teachers in health education courses, whereas the final phase of the intervention included sports equipment that could be used during class		Proportion of excessive users, proportion of those gaming or spending time on the internet for ≥2 hours a day, and proportion of those being on the internet overnight	
		**Online**							
			Brailovskaia et al [12], 2022	RCT of 619 young adult smartphone users (at least 75 minutes per day) in Germany		Abstinence vs reduction of smartphone use by 1 hour (vs control)		Daily smartphone use and problematic smartphone use	
			Ko et al [9]^h^, 2021	Controlled pretest-posttest study of 16 young adults who perceived themselves as social media addicts		Social Media Addiction Coach		Social media use	
			Thai et al [10], 2023	RCT of 260 (220 completed) college students who regularly used social media (>45 minutes per day) in Canada who had symptoms of depression or anxiety		IG^i^ was asked to limit social media use to 1 hour per day for 3 weeks		Smartphone use	
**Pretest-posttest studies without control groups**									
	**Primary prevention**								
		**Education**							
			Chau et al [45], 2019	Before-and-after study of 248 primary school students in Hong Kong, China		Wise IT use program		Distribution of normal, at-risk, and high-risk gamers and levels of IGD symptoms	
			Ke and Wong [46], 2018	Pretest-posttest study of 45 teenage students from government secondary schools in Malaysia		PIP-IU-Y^j^		PIU^k^	
			Ke and Wong [47], 2018	Pretest-posttest study of 157 teenage students from government secondary schools in Malaysia		PIP-IU-Y		PIU	
			Kent et al [48], 2021	Pretest-posttest study of 10 undergraduate students in the United Kingdom		An intervention based on the stages of change model was delivered via a smartphone app		Problematic mobile phone use and internet addiction measured by IAT^l^	
		**Online**							
			King et al [49], 2017	27 young adult MMO^m^ players		Participants were asked to refrain from gaming over a single weekend		Gaming hours and IGD symptoms	
	**Secondary prevention**								
		**Community**							
			Männikkö et al [50], 2022	Pretest-posttest study of 37 young adults recruited from 5 cities in Finland who self-reported excessive gaming		The Limitless Gaming Bootcamp		Problematic gaming, gaming time, internet use, and television viewing time	
		**Other**							
			Heffler et al [51], 2022	Pretest-posttest study of 9 preschool-aged children diagnosed with autism who viewed screens for at least 2 hours a day		Parents viewed a 40-minute educational video and then received weekly 1-hour in-home support visits from a trained therapist		Parents’ reports of children’s screen time and adverse effects of keeping screens off	
**Observational studies and protocols**									
	**Primary prevention**								
		**Education**							
			Barker [52], 2021	Protocol only; program will target preadolescent students in the United States who play internet games		Mindfulness and DBT^n^ group psychoeducation and skill building to prevent problematic internet gaming		—^o^; protocol only	
			Hansen et al [53], 2021	A cross-sectional survey conducted with 542 “multipliers” who had been trained across Germany to deliver the program		“Net-Piloten” (Net Pilots) peer project for the prevention of social media addiction		Implementation of the intervention, barriers, and potential solutions	
			Neverkovich et al [31]^p^, 2017	Experimental study of 657 high school, college, and medical school students in Moscow and Irkutsk, Russia		Psychological and educational support programs providing motivational, cognitive, practice-oriented, and reflective units		Internet addiction and social network addiction	
			Tang et al [54], 2021	Protocol for a cluster-randomized RCT with a proposed sample of 240 level-5 primary school students in Hong Kong, China		MBCP^q^ aimed at improving adolescents’ resilience, changing their smartphone use behavior, and reducing smartphone addiction symptoms		—; protocol only	
		**Community**							
			Krossbakken et al [55], 2018	A posttest-only experimental study of 1762 (1657 analyzed) guardians of young children (aged 8-12 years) in Norway		The intervention consisted of a single mailed brief parental guide on “how to regulate video game behavior in children”		Gaming time and video game problems (IGD criteria)	
			Sela [56], 2021	Protocol only		Training and assistance in setting parental controls (TPM^r^) vs training in PVC^s^ vs both (TPM+PVC)		—; protocol only	
		**Online**							
			Hayes and Jones [57], 2022	Protocol only; the study was terminated due to inability to recruit and retain sufficient participants		A 6-session web-based group intervention targeting problematic smartphone use		—; study was terminated	
			Schmuck [58], 2020	Cross-sectional web-based survey of 500 young adults in Austria who used a smartphone		Self-reported existing use of one of several digital detoxification apps for smartphone, such as iOS Screen Time, Android Digital Wellbeing, Moment, Forest, QualityTime, Detox, Space, Offtime, RealizeD, or similar		Problematic smartphone use	
		**Other**							
			Choi et al [32], 2018	Panel survey of a representative sample of 243,957 middle and high schoolers in South Korea		Article 26 of the Juvenile Protection Act (shutdown policy): “Internet games should not be offered to those under the age of 16 from 12:00 am to 6:00 am”		Problematic use (>300 minutes per day, called “addiction” in the study) and weekly internet use	

^a^RCT: randomized controlled trial.

^b^IGD: internet gaming disorder.

^c^Excluded from vote counting because of lack of direct comparison between the intervention and control groups.

^d^DWB-S: Digital Well-Being–Schools.

^e^GOI: Game Over Intervention.

^f^CBT: cognitive behavioral therapy.

^g^PROTECT: Professioneller Umgang mit technischen Medien (Professional Use of Technical Media).

^h^Excluded from vote counting because of unclear analysis reporting (direction of effect unclear).

^i^IG: intervention group.

^j^PIP-IU-Y: Psychological Intervention Program–Internet Use for Youth.

^k^PIU: problematic internet use.

^l^IAT: Internet Addiction Test.

^m^MMO: massively multiplayer online game.

^n^DBT: dialectic and behavioral therapy.

^o^Not applicable.

^p^Excluded from vote counting due to unclear study design and analysis.

^q^MBCP: mindfulness-based cognitive program.

^r^TPM: Technological Parental Monitoring.

^s^PVC: Parental Vigilant Care.

### Outcomes

#### Overview

Outcomes were assessed using a variety of validated and ad hoc measures related to problematic use, including validated scales (eg, Internet Addiction Test [43]), subscales or questions from validated scales (eg, “two sub-dimensions of disturbance of adaptive functions and withdrawal from the Smartphone Addiction Scale” [13]), modified scales (eg, “a modified version of the brief Bergen Social Media Addiction Scale” [12]), and ad hoc questions (eg, “Technology-Related Parenting Strategies” [41]). Parent or school counselor reports were sometimes used for recruitment and eligibility criteria or for outcome measurement. No review limited measures to specific scales.

#### Synthesized Findings

##### RQ 1: What Health Promotion and Prevention Interventions Have Been Used to Promote Digital Well-Being and Prevent Problematic Digital Media and Technology Use in Children, Adolescents, and Youth?

This section describes the level of prevention, sector of implementation, and behavior or symptom targets. In total, 60% (21/35) of the primary studies described primary or universal prevention interventions, 37% (13/35) described secondary or selective prevention approaches, and 3% (1/35) of the studies [49] targeted both primary and secondary prevention. Of the 21 studies describing primary interventions, 17 (81%) were conducted in educational settings, 2 (10%) were conducted in community settings, 1 (5%) was online, and 1 (5%) was a national policy. Of the 13 secondary intervention studies, 8 (62%) were conducted in educational settings, 1 (8%) was conducted in a community setting, 1 (8%) was conducted in a health care setting, 2 (15%) were online, and 1 (8%) was unclear. The study targeting both primary and secondary prevention was online [49]. Of the 6 reviews, 1 (17%) included only universal or primary prevention studies [72], 1 (17%) included universal and selective or secondary prevention studies [73], and 1 (17%) included universal and indicated prevention studies [5]; 3 (50%) reviews did not indicate a prevention level.

**Figure 2:**
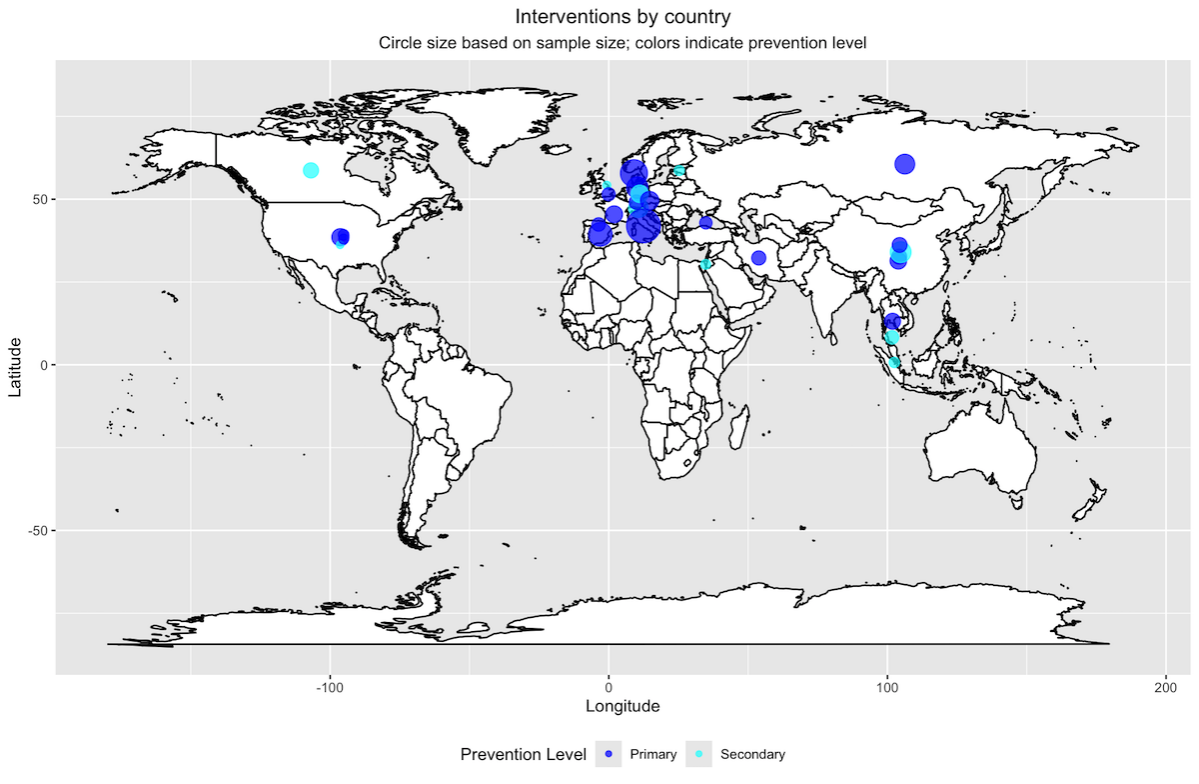
Sample size and prevention level by country. This figure does not include a national study of >200,000 adolescents in South Korea, as this represents an outlier value that makes the visualization of other sample sizes impossible.

Figure 2 illustrates the sample sizes by country and prevention level for all primary studies except a study of South Korea’s Juvenile Protection Act [32], which had 243,957 participants.

Sample sizes ranged from 32 to 243,957 (mean 13,386.42, SD 55,839.74; median 362) for primary prevention studies and 9 to 775 (mean 216.36, SD 272.75; median 45) for secondary prevention studies. A total of 67% (4/6) of the primary prevention studies with >1000 participants took place in Europe. Of the 9 studies with <100 participants, 5 (56%) were delivered by health care professionals or school counselors, and 3 (33%) were self-administered (eg, apps or online abstinence interventions). Only 6% (2/33) of the interventions were delivered without interaction with the target population (or teachers and parents): South Korea implemented the Juvenile Protection Act, which was designed to limit internet access for adolescents [32], and a study in Norway [55] mailed a brief parental guide with advice and strategies for regulating children’s video gaming.

Various behaviors and problems associated with digital well-being were targeted as outcomes of the interventions. Internet use and game use were the most common intervention targets—39 outcomes were measured across 20% (7/35) of the studies for internet use, and 33 outcomes were measured across 29% (10/35) of the studies for game use. All reviews (Table 2) defined intervention targets regarding behaviors and addictions broadly. The reviews generally synthesized literature on screen time (4/6, 67%), although 17% (1/6) of the reviews examined prevention of internet addiction, and 17% (1/6) focused on abstinence interventions for various behavioral addiction outcomes [5,74].

**Table 2:** Characteristics of the included reviews

Review	Review type, aims, and population	Studies; participants^a^, N	Results	Conclusions	Study quality criteria met; AMSTAR 2^b^ rating
Fernandez et al [74], 2020	Systematic review of short-term abstinence interventions across potential behavioral addictions, including benefits or counterproductive consequences of abstinence	47; 8245^c^	Voluntary abstinence may be useful for specific problematic behaviors, especially gaming, pornography use, mobile phone use, and social media use	The paper describes the benefits of short-term abstinence but also discusses that harms were not addressed systematically in the studies. There were issues of reporting, including quality review (eg, outcome-level limitations were not addressed) and outcome reporting (it was unclear how “key findings” were selected for inclusion in the review).	No; critically low
Jones et al [73], 2021	Systematic review and meta-analysis of behavioral interventions to reduce children’s screen time	204; 162,494^c^	The review found a small positive effect of interventions, with interventions targeting younger children and interventions with a shorter duration showing larger effects	This high-quality meta-analysis found overall benefits of screen time interventions and was systematic and complete about reporting how outcomes were chosen and describing limitations and other study quality criteria. However, potential harms were not examined. The authors note the importance of “determining the active ingredients to optimize interventions along the translational continuum.”	Yes; critically low
Krafft et al [75], 2021	Systematic review of intervention strategies to reduce screen time among children from birth to 12 years of age	11; 5627^c^	Interventions can promote awareness of and sometimes reduce screen time	The findings were not consistent, and screen time “awareness” was sometimes described as a benefit. The paper lacked a clear description of how outcomes would be reported and did not discuss limitations at the outcome level or potential harms.	No; critically low
Oh et al [72], 2022	Systematic review and meta-analysis of interventions to promote healthy screen time and reduce sedentary behavior in school-aged children and adolescents in all settings	51; 16,418	The review found moderate quality of evidence for reduction in television viewing but low quality of evidence for overall screen time and no effects for gaming and studies with quasi-experimental designs.	This high-quality review showed the benefits of nondigital screen time interventions, particularly for television watching. The review did not address harms or discuss limitations at the study level but did systematically address bias and quality by grading the evidence.	Yes; critically low
Throuvala et al [5], 2019	Systematic review of prevention programs for internet addiction within the school context	20; 226,762^c^	This review highlighted the diversity in program scope and outcomes and mixed results in reducing internet and gaming use.	The review found very diverse interventions and results, drawing no overall conclusions as to the benefits or lack thereof. The review did not describe how outcomes would be reported, assess bias or quality, discuss harms, or address limitations other than at the study level.	No; critically low
Throuvala et al [71], 2021	Systematic review of intervention strategies to reduce screen time among adolescents	15; 17,241^c^	Interventions are more likely to reduce excessive screen time if they include strategies targeting other factors that drive internet use behaviors and consider activities separately rather than lumping them together in a single “screen time” construct.	The review highlighted the overall mixed effectiveness of the studies and the way in which screen time behaviors were measured in interventions aiming to reduce sedentary behavior and pointed out appropriately that interventions need to consider content, context, and reasons for use rather than solely assessing changes in amount of screen time. It did not evaluate harms from the interventions or describe how outcomes were chosen.	No; critically low

^a^The number of included studies in a review was taken from the review’s PRISMA (Preferred Reporting Items for Systematic Reviews and Meta-Analyses) flow diagram (where possible) or from reports in the text or tables of each review.

^b^AMSTAR 2: A Measurement Tool to Assess Systematic Reviews-2.

^c^The total number of participants was calculated from a summary table or from the included studies.

##### RQ 2: What Theoretical or Treatment Models and Approaches Inform the Development of These Interventions?

**Figure 3:**
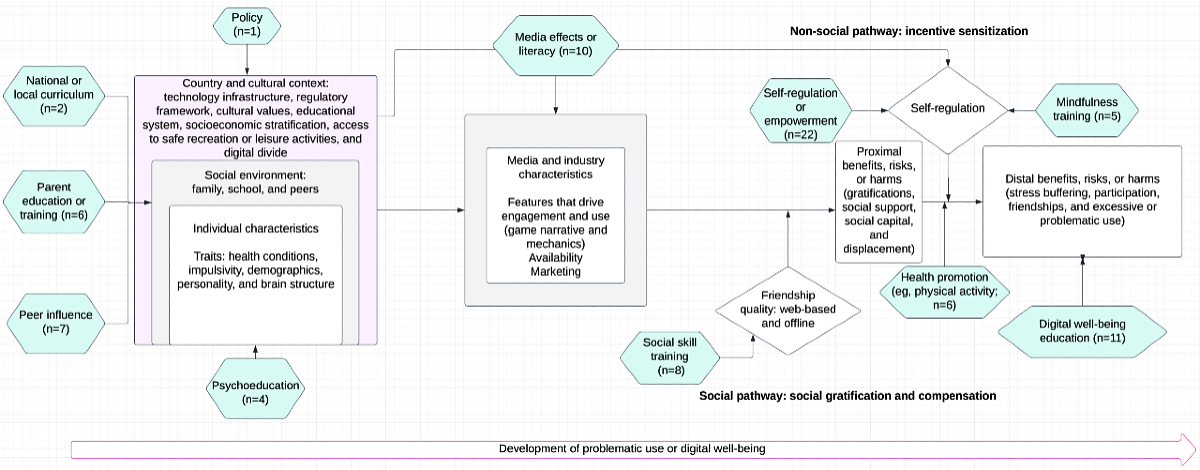
Development of problematic digital media use and digital well-being (DWB) with mapped intervention approaches.

Characteristics and approaches used in interventions were usually described in tables or appendices, but some studies reported information in a way that was difficult to interpret. We extracted intervention approaches and mapped these to our proposed conceptual framework (Figure 3), resulting in 11 mapped intervention approaches.

For example, we mapped the intervention approaches “gain more knowledge about gaming addiction and its effects” [33] and “raise awareness about the consequences of excessive use of video games on sleep, school investment, and family” [35] to digital well-being education (ie, education on the potential long-term problems and benefits of digital media or technology use) but mapped modules focusing on mental health symptoms, including “learning ways to reduce social anxiety” [47], to psychoeducation. Interventions including a self-regulation approach were most prevalent (22/33, 67%) and included aims related to regulation and time management directly [30,33,38].

Of the 33 interventions described in the 35 included studies, most (26/33, 79%) combined approaches. The most common components focused on education or training components, such as education about self-regulation, digital well-being education, media literacy and effects, or mental health symptoms and coping (psychoeducation). Combined interventions such as Net Pilots [8] focused on educating about self-regulation of digital technology use through critical examination of one’s own use but were also delivered by peer “net pilots” (peer influence). Most interventions that combined approaches used 2 approaches (11/26, 42%), followed by 3 approaches (6/26, 23%) and 4 approaches (5/26, 19%). The most commonly combined intervention approaches were self-regulation and digital well-being education, followed by self-regulation and media literacy and effects. Interventions with a single approach were most often delivered via mobile phone and encouraged temporary abstinence from or reduction of social media or mobile phone use. Other single-approach interventions included an informational pamphlet to help parents promote healthy gaming among their children [55], a study of a national policy in South Korea [32], a protocol for a study comparing various types of parent training [56], and a protocol for a study of a mindfulness-based intervention [10].

Most review articles (4/6, 67%) specifically addressed intervention components that might promote effectiveness, looking for “active ingredients” associated with success [73]. The detailed meta-analysis by Jones et al [73] aimed to “...identify the behavior change techniques and study characteristics associated with effectiveness in behavioral interventions to reduce children’s (0-18 years) screen time.” This review used the Behavior Change Technique Taxonomy by Abraham and Michie [76] to analyze studies by behavior change technique, finding that interventions that included goals, feedback, and planning were more likely to be effective and that interventions delivered within less than a year and with smaller samples had larger effects than those delivered over a longer period or that had larger samples. Other reviews described intervention components, methods, and frameworks in tables or text.

##### RQ 3: How Effective Are These Interventions When Compared to Other Interventions or No Intervention?

To determine the effectiveness of the interventions, we extracted relevant outcome data from 19 controlled trials (ie, RCTs and controlled before-and-after studies), as described previously, and then conducted vote counting for controlled trials of interventions that directly compared an intervention and a control group (14/19, 74%). A total of 71% (10/14) of the studies took place in the education sector, whereas 29% (4/14) of the studies were online. None of the included controlled studies took place in health care, the community, or a policy setting. No controlled trials delivered interventions to children only. Due to the differing characteristics of the interventions, their different target and delivery populations, and the rapid nature of our review, we did not synthesize outcomes quantitatively or conduct subgroup analyses. However, data are available on our OSF website.

Table 3 presents results for the 14 included controlled trials that examined our outcomes of interest. Of the 51 outcomes comparing intervention and control groups, most measured gaming time (n=14, 27% of the outcomes across 5/14, 36% of the studies). Internet use time, problematic gaming, and problematic internet use accounted for 12% (6/51) of the outcomes each. Interventions delivered to study populations other than young people (eg, parents or teachers) had, in general, longer follow-up periods (up to 3 months after baseline), although one intervention delivered to adolescents and youth [30] had follow-ups at 1, 4, and 12 months after the intervention.

Vote counting showed that, of the 51 outcomes, 44 (86%) favored the intervention group. Under the null hypothesis of no difference between groups, the probability of observing this result would be <0.001. The estimated Jeffrey 95% CI for this proportion ranged from 0.75 to 0.94, supporting improvements in digital well-being. Of those comparisons favoring the control group (7/51, 14%), 86% (6/7) reflected likely baseline differences [30,37]. Just 1 outcome from 14% (1/7) of the studies that favored the control group had an absence of apparent baseline differences [30].

Effects on minutes of daily use varied widely. The minimum absolute difference in mean minutes after the intervention was 2.6 (favoring the control group), whereas the maximum absolute difference was 110.5. The average difference in minutes per day for all interventions was 22.5. Considering only RCTs (10/15, 67%) made little impact—the average reduction in minutes of use across platforms fell to 18.6, and the range remained the same. The lowest impacts were found for gaming time and screen time, which showed an average reduction of 18.9 minutes per day (12 outcomes across 5/15, 33% of the studies for gaming time and 4 outcomes across 2/15, 13% of the studies for screen time). In contrast, interventions measuring time on social media showed an average reduction of 40.9 minutes per day (4 outcomes across 4/15, 27% of the studies), whereas interventions measuring smartphone use time showed an average reduction of 34.0 minutes per day, and those measuring internet use time showed an average reduction of 25.1 minutes per day.

Harms or unintended consequences of an intervention were not discussed in any of the assessed primary studies. One review [74] discussed harms and found that, generally, there was a lack of discussion about the potential adverse effects and unintended consequences of interventions (eg, rebound effects and more harmful compensatory behaviors) in reports of primary studies (see the OSF website [59], review study data extraction).

A total of 33% (2/6) of the reviews analyzed intervention effects using statistical analyses. A meta-regression of 204 studies [73] found a small positive effect of interventions to reduce screen time, with interventions including goals, feedback, and planning having slightly higher effect sizes (overall standard difference of the mean=0.116; interventions including goals, feedback, and planning=0.145). The other meta-analysis [72] found an overall small reduction in television viewing time (12.45 minutes) for nondigital interventions versus control groups in 23 studies with moderate quality of evidence but high uncertainty in effects on minutes per day of computer or video gaming screen time or screen time overall, with mean differences ranging from approximately 5 minutes to <30 minutes (Multimedia Appendix 1 [8-13,30-58]).

**Table 3:** Controlled studies by outcome type and target_a_.

Study and outcome					Study type		Participants, N		Time point of assessment		Intervention group value		Control group value		Statistical test results		*P* value
**Children and adolescents combined**																	
	**Time using media**																
		**Gaming time**															
			Hansen et al [8], 2022: minutes per weekday	CS^b^		210		After the intervention		143		166		*F*_1832_=6.45		.01	
			Hansen et al [8], 2022: minutes per weekend	CS		210		After the intervention		209		240		*F*_1832_=4.06		.04	
		**Internet use time**															
			Hansen et al [8], 2022: minutes per weekday	CS		210		After the intervention		102		121		*F*_1832_=5.83		.02	
			Hansen et al [8], 2022: minutes per weekend	CS		210		After the intervention		152		180		*F*_1832_=6.73		.001	
		**Social media use time**															
			Hansen et al [8], 2022: minutes per weekday	CS		210		After the intervention		74		87		*F*_1832_=2.97		.09	
			Hansen et al [8], 2022: minutes per weekend	CS		210		After the intervention		99		121		*F*_1832_=4.08		.04	
	**Problematic use**																
		**Problematic gaming**															
			Ortega-Barón et al [40], 2021: IGDS9-SF^c^	CS		165		After the intervention		3.88		5.56		*F*=5.39		<.05	
		**Problematic internet use**															
			Hansen et al [8], 2022: CIUS^d^	CS		210		After the intervention		—^e^		—		*F*_1832_=14.76		.001	
			Ortega-Barón et al [40], 2021: GPIUS2^f^	CS		165		After the intervention		16.71		25.83		*F*=8.29		<.01	
**Adolescents and youth**																	
	**Time using media**																
		**Gaming time**															
			Bickham et al [34], 2018: minutes after school	CS		412		After the intervention		22		42.5		*F*_1460_=4.8		.03	
			Bickham et al [34], 2018: minutes on Saturday	CS		412		After the intervention		33.5		42.9		*F*_1463_=0.8		.37	
			Bonnaire et al [35], 2019: minutes per weekday	CS		384		After the intervention		119.8		188.9		*F*_2_=18.57		<.001	
			Bonnaire et al [35], 2019: minutes per weekend	CS		384		After the intervention		177.8		213.1		*F*_2_=31.06		<.001	
		**Internet use time**															
			Bickham et al [34], 2018: minutes after school	CS		412		After the intervention		36.4		65.8		*F*_1460_=2.9		.09	
			Bickham et al [34], 2018: minutes on Saturday	CS		412		After the intervention		42.3		55		*F*_1459_=4.7		.03	
			Bonnaire et al [35], 2019: minutes per weekday	CS		384		After the intervention		300.2		325.5		*F*_2_=17.68		<.001	
			Bonnaire et al [35], 2019: minutes per weekend	CS		384		After the intervention		317.9		353.1		*F*_2_=6.90		.001	
		**Screen time**															
			Bickham et al [34], 2018: minutes after school	CS		412		After the intervention		49		82.2		*F*_1459_=28.1		<.001	
			Bickham et al [34], 2018: minutes on Saturday	CS		412		After the intervention		63.3		78.3		*F*_1457_=6.1		.01	
		**Social media use time**															
			Thai et al [10], 2023	RCT^g^		260		After the intervention		78.25		188.76		*F*_3648_=94.05		<.001	
			Throuvala et al [11], 2020	RCT		143		After the intervention		130.2		148.2		*F*_1, 140_=3.697		.06	
		**Smartphone time**															
			Brailovskaia et al [12], 2022	RCT		619		1 month		157.43		198.2		*F*_4, 1187_=6.754		<.001	
			Brailovskaia et al [12], 2022	RCT		619		4 months		162.02		187.33		*F*_4, 1187_=6.754		<.001	
			Throuvala et al [11], 2020	RCT		143		After the intervention		210.6		246.6		*F*_1, 140_=4.43		<.001	
	**Problematic use**																
		**Problematic gaming**															
			Bonnaire et al [35], 2019: GAS-7^h^ (proportion of gamers screening positive for IGD^i^)	CS		384		After the intervention		10		23		Not given		.03	
		**Problematic internet use**															
			Affouneh et al [43], 2021: IAT^j^	RCT		30		After the intervention		2.83		3.88		*F*_1_=304.44		<.001	
			Lindenberg et al [30], 2022: modified CSAS^k^	RCT		422		1 month		*14.46* ^l^		*12.48* ^l^		γ_11_=–0.128		.03	
			Lindenberg et al [30], 2022: modified CSAS	RCT		422		4 months		12.09		12.74		γ_11_=–0.128		.03	
			Lindenberg et al [30], 2022: modified CSAS	RCT		422		12 months		9.2		10.07		γ_11_=–0.128		.03	
		**Problematic social media use**															
			Throuvala et al [11], 2020: Bergen SMAS^m^	RCT		143		After the intervention		15.12		17.24		*F*_1, 140_=6.96		<.001	
			Weaver [41], 2021: SMUQ^n^	CS		54		After the intervention		15.6		22		*F*_2, 52_=6.02		<.05	
		**Problematic smartphone use**															
			Brailovskaia et al [12], 2022: modified Bergen SMAS	RCT		619		After the intervention		*12.8* ^o^		*12.14* ^o^		*F*_6, 1806_=8.40		<.001	
			Brailovskaia et al [12], 2022: modified Bergen SMAS	RCT		619		1 month		11.22		12.28		*F*_6, 1806_=8.40		<.001	
			Brailovskaia et al [12], 2022: modified Bergen SMAS	RCT		619		4 months		11.4		12.16		*F*_6, 1806_=8.40		<.001	
**Other**																	
	**Time using media**																
		**Gaming time**															
			Apisitwasana et al [33]^p^, 2018: days per week	RCT		310		After the intervention		2.83		3.44		—^q^		<.001	
			Apisitwasana et al [33], 2018: days per week	RCT		310		3 months		3.57		4.03		—		<.05	
			Apisitwasana et al [33], 2018: minutes per weekday	RCT		310		After the intervention		54.6		64.8		—		<.05	
			Apisitwasana et al [33], 2018: minutes per weekday	RCT		310		3 months		72.6		84.6		—		NS^r^	
			Apisitwasana et al [33], 2018: minutes per weekend day	RCT		310		After the intervention		103.8		118.2		—		NS	
			Apisitwasana et al [33], 2018: minutes per weekend day	RCT		310		3 months		129		146.4		—		NS	
			Li et al [37]^s^, 2019 (parent report)	RCT		362		After the intervention		*45.2* ^o^		*32.6* ^o^		*F*_1.72, 516.22_=4.86		.01	
			Li et al [37], 2019 (parent report)	RCT		362		3 months		*38.8* ^o^		*36.3* ^o^		*F*_1.72, 516.22_=4.86		.01	
		**Screen time**															
			Sanders et al [42], 2018: parental assessment of daily time	RCT		32		After the intervention		*180.6* ^o^		*160.8* ^o^		Not conducted		N/A^t^	
			Sanders et al [42], 2018: parent diary assessment of daily time	RCT		32		After the intervention		133.8		181.2		Not conducted		N/A	
		**Social media use time**															
			Gui et al [13]^u^, 2023: interactive use index	RCT		2997		After the intervention		28.8		30.5		*t*=–1.7		>.05	
	**Problematic use**																
		**Problematic gaming**															
			Apisitwasana et al [33], 2018: GAST^v^	RCT		310		After the intervention		9.34		17.1		*F*_1, 823_=7.79		.001	
			Apisitwasana et al [33], 2018: GAST	RCT		310		3 months		11.48		20.57		*F*_1, 823_=7.79		.001	
			Li et al [37], 2019: parent version of adapted KSAS^w^	RCT		362		After the intervention		*25.48* ^o^		*24.44* ^o^		*F*_1.93, 520.5_=1.27		.28	
			Li et al [37], 2019: parent version of adapted KSAS	RCT		362		3 months		*25.05* ^o^		*24.61* ^o^		*F*_1.93, 520.5_=1.27		.28	
		**Problematic smartphone use**															
			Gui et al [13], 2023: disturbance	RCT		2997		After the intervention		34.2		36.2		*t*=–2.0		<.01	
			Gui et al [13], 2023: withdrawal	RCT		2997		After the intervention		27.3		28.5		*t*=–1.3		>.05	

^a^The table describes only controlled trials, excluding subgroup analyses, that had estimates after the intervention or at a follow-up time point. Intervention and control values represent predicted or observed means, totals, or proportions as reported by the authors of the primary studies. Values for time are shown in minutes per day, either given or converted from other measures, unless otherwise specified.

^b^CS: other controlled study.

^c^IGDS9-SF: Internet Gaming Disorder Scale—Short-Form.

^d^CIUS: Compulsive Internet Use Scale.

^e^Not reported.

^f^GPIUS2: Generalized Problematic Internet Use Scale.

^g^RCT: randomized controlled trial.

^h^GAS-7: Game Addiction Scale-7 (item).

^i^IGD: internet gaming disorder.

^j^IAT: Internet Addiction Test.

^k^CSAS: Video Game Addiction Scale.

^l^Studies favoring the control group in the absence of baseline differences.

^m^SMAS: Social Media Addiction Scale.

^n^SMUQ: Social Media Use Questionnaire.

^o^Studies favoring the control group in the presence of baseline differences.

^p^This intervention was delivered to both children and parents.

^q^Not reported.

^r^NS: the authors of the study reported significant *P* values with asterisks in the manuscript, and these comparisons were not reported with asterisks.

^s^This intervention was delivered to parents.

^t^N/A: not applicable.

^u^This intervention was delivered to teachers.

^v^GAST: Game Addiction Screening Test.

^w^KSAS: Korean Internet Addiction Scale for Adolescents, adapted to fit the video gaming context.

##### RQ 4: What Other Characteristics of Intervention Setting or Delivery May Limit or Promote Translation to Other Contexts?

To synthesize evidence in a way that might inform translation to other contexts, we used the CASP tools to capture data about study quality and intervention implementation. In this review, some studies (7/35, 20%) included information about factors important to implementation, such as attrition and dropout rates [50], participant adherence [12,43,51], and fidelity [13,42,53]. One intervention [48] reported 100% participant engagement with no attrition. Others (3/35, 9%) evaluated the perceived competence of the person delivering the intervention [42] or success in implementation in the school setting [13,53]. However, most studies (22/35, 63%) provided little or no information on implementation or fidelity.

Our review noted several other characteristics of the studies that could affect the ability to translate the findings to other contexts. First, we found considerable diversity in availability of information about costs and funding sources. One protocol [52] calculated the cost of their intervention in a very detailed manner; however, such data were absent for all other studies. Studies disclosed varying sources of financial support, including support from national governments [9,13,31], public health organizations [49,53], charitable foundations [48,49], and other research organizations [37,45,51].

The availability of intervention manuals is also an important factor for translation. Few studies (3/35, 9%) used an intervention manual, and descriptions of the interventions were sometimes lacking. Some studies (5/35, 14%) incorporated a table with the elements and purpose for each module in either the body of the paper or an appendix. In contrast, some studies (2/35, 6%) made explicit references to the use of intervention manuals, such as a “gaming addiction prevention manual” for parents [33] or “manual-based CBT intervention” [47].

Factors contributing to scalability and resource use were often more clearly described and varied widely. For example, one intervention [51] used weekly 1-hour one-to-one parent visits by a therapist to promote parental restrictions on screen time and improved social interactions between parents and preschoolers with autism. Other interventions (17/35, 49%) were delivered by (or included) psychologists, school counselors, or school prevention officers [13,30,35,43,47,50] or teachers [13,33,34,40,54], usually over many weeks. Some interventions were delivered by peers with or without a professional colead, such as the Net Pilots intervention by Hansen et al [8,53], which was delivered by adolescents, or the joint peer coach (“a young adult volunteer with a personal history of problematic gaming”) and therapist-delivered intervention by Männikkö et al [50]. In contrast, some interventions (7/35, 20%) were online, requiring no direct interaction between intervention providers and participants, including planned abstinence interventions [12,49] or interventions using apps [9,11].

The reviews provided some information about study and intervention elements that could promote translation, such as intervention intensity, components, and approaches. For example, Jones et al [73] found larger effect sizes for interventions with smaller sample sizes and shorter durations. However, no review discussed funding sources for interventions, and only 17% (1/6) of the reviews systematically examined harms, making it difficult to evaluate the costs, risks, and benefits of implementation in different contexts.

##### RQ 5: What Quality Issues Should Be Addressed in Future Empirical Studies of These Interventions?

Using the analytical questions of the CASP framework for both controlled trials and observational studies (see the OSF website), we found several design- and reporting-related issues. Most studies (32/35, 91%) reported information about PICOS with a few exceptions, notably, 6% (2/35) of the studies, which did not provide details on the population studied and the comparator [36,47], and 3% (1/35) of the studies, which lacked a clear RQ and intervention description [31]. Second, many of the included experimental and quasi-experimental studies did not report adequate detail about randomization (12/27, 44%) and blinding (7/27, 26%). Third, not all studies (15/27, 56%) addressed loss to follow-up clearly, and power calculations were rare (4/27, 15%).

Of the 6 reviews, only the 2 (33%) meta-analyses clearly specified which outcomes would be extracted from the studies. Other reviews did not specify how they would select outcomes, using language such as “critical outcomes” [71] and “key outcome measures” [74], resulting in a designation of low reliability according to our prespecified criteria. In addition, all reviews received an AMSTAR 2 score of “critically low” (see the OSF website), with no reviews reporting on funding sources of individual studies and few (4/35, 11%) reporting preregistered protocols or duplicate data extraction.

## Discussion

### Principal Findings

We conducted a rapid systematic review of the recent literature (since 2017) to investigate the current evidence on prevention approaches to promote digital well-being, defined as the healthy use of digital media and technology and the absence of problems resulting from excessive use, among children, adolescents, and youth. The final selection included 41 studies (n=35, 85% primary studies and n=6, 15% reviews). In the primary studies, internet and game use were the most common outcomes, and most interventions (22/33, 67%) included education about self-regulation. Digital well-being education, media literacy and effects, or mental health symptoms and coping (psychoeducation) were other common intervention approaches. Most interventions (26/33, 79%) also combined several approaches. Vote counting of controlled trials showed benefits for all interventions. A total of 33% (2/6) of the reviews conducted statistical analyses and found that the interventions studied had a small but beneficial impact on decreasing the amount of time that people spent on screens.

Similar to previous reviews in this area [5,17], this review also found that most programs for universal and selective intervention to promote digital well-being and prevent problematic technology use were implemented in educational settings. Many of these interventions can be classified as complex interventions that contain multiple active ingredients and tap into different potential behavior change strategies. Intervention approaches that aimed at improving the self-regulation of participants were the most common in the included studies, and this self-regulation closely resembled goals, feedback, and planning in a widely used taxonomy of behavior change strategies [76]. However, a close investigation of the individual studies showed that self-regulation, even if mentioned using that term, could look very different. For example, the 6-week intervention by Apisitwasana et al [33] contained 5 weeks of instruction specific to self-regulation, whereas the study by Affouneh et al [43] delivered 1 week of “self-discipline” instruction for avoidance of addictions specifically, with other weeks targeting “problem solving strategies for dealing with problems to prevent addictive behaviors” and “effective use of time to prevent addictive behaviors.” While grouping of intervention approaches is important for systematic investigation, these limitations in comparability of approaches across studies should be kept in mind, and detailed reporting of intervention approaches in future studies is needed.

Moreover, the interventions included in this review varied widely in intensity, with some interventions requiring a single session and others requiring 1:1 weekly clinician visits over weeks. Peer-delivered interventions may reduce overall costs. These have been successful for other addictions and are a common method of task sharing for psychosocial interventions, especially in middle-income settings [77]. However, this depends on the intensity of training; if extensive training of teachers is needed for them to be able to train peers and teenage peers then age out, these extensive trainings may need to be repeated with high frequency. Although our review was not designed to provide a quantitative evaluation of the studies, the included review by Jones et al [73] found that interventions with shorter durations were more likely to be effective in reducing screen time. Interventions with shorter durations are more likely to be feasibly implemented, and future studies should aim to examine associations among intervention intensity, feasibility, and efficacy.

Harms are not usually assessed in the reporting of studies of interventions for behavioral addictions [74], but it is important for digital well-being. Interventions involving abstinence from addictive behaviors may result in rebound effects after the intervention or may lead to engagement in compensatory behaviors that could be more harmful (eg, searching for gaming-related pornography when restricted from gaming [78]). We found that some comparisons included in our vote counting (7/51, 14%) had results that favored the control group; however, these were usually due to baseline differences (6/7, 86%). In contrast, individuals struggling with more harmful addictive behaviors such as alcohol or drug use may use digital behaviors—even excessive ones—as a less harmful compensatory behavior to deal with cravings and withdrawal symptoms [79]. Findings on both the potential benefits and negative consequences of digital well-being interventions underscore the need for RCTs with appropriate treatment of any baseline differences as well as comprehensive and consistent reporting of outcomes in future research.

As suggested by other scholars [17], our review included an explicit aim to report aspects of published work that impact how the findings can be translated and applied to different contexts (eg, countries and areas of the world that are starting to see technology use problems but have not developed or systematically evaluated digital well-being interventions themselves). Many studies included in this review (20/35, 57%) were conducted in Europe and the Global North. Whether these studies can translate to other contexts is another question. Our review focused on identifying factors that can help interventionists decide whether an intervention will work in their setting. Things to consider are information on intervention costs, intervention manuals, and information on scalability. Only a minority of studies (7/35, 20%) reported some of these aspects. Future studies would benefit from including greater detail on factors that would help inform translation to other contexts.

Moreover, formulating a well-defined RQ; using a clear study design; and providing comprehensive information about the studied population, intervention, comparator, and measured outcomes are crucial for understanding study quality. Reporting guidelines for empirical studies and systematic reviews provide clear recommendations for elements of study design, methods, and outcomes to report and should be used in future work. We hope that, by explicitly focusing on these topics, our review can inspire future studies to report this crucial information and, thus, make a contribution to developing the international evidence base in this important and growing area of research.

### Limitations

This review has some limitations. As this was designed to be a rapid review, we deferred some elements that are common in other types of reviews (eg, double data extraction, contacting authors for missing data, performing meta-analyses or otherwise evaluating effect sizes, and conducting a formal risk-of-bias assessment [64]). Moreover, we included the most recent literature published since 2017, which is appropriate for a rapid review and quickly changing technology platforms and use patterns. We excluded studies that described “treatment” of “individuals with disorder” even if the screening instrument scores of the included samples fell below the cutoffs used for the disorders. Thus, we may have missed other interventions that could be helpful to improve digital well-being. Although we searched 5 databases, we missed interventions such as the Digital Balance survey and website and Ithra Sync, which were delivered through websites and social media and did not show up in our searches [16,80]. Outcome-level limitations include that we did not limit our review to studies with specific outcome measures or scales or even to studies with validated outcome measures. This allowed us to include a wide range of studies but runs the risk of including weak outcome measures (eg, inconsistent measures for the same outcome across the studies and problems with self-reporting screen time). At the study level, we found that poor reporting of key information, including fidelity of intervention implementation or missing data, presented significant limitations to our ability to synthesize data into evidence. In some cases, study design and statistical analysis were difficult to ascertain [44] or missing [31]. Only a subset of RCTs (14/19, 74%; Table 3) were included in vote counting because they reported direct comparison between the intervention and control groups on the outcomes selected for this review. In addition, the dates of our review, 2017 to 2023, included the 3 years of the COVID-19 pandemic (ie, March 2020 to May 2023), making it possible that empirical studies occurring during this time could contain cohort effects. Finally, most reviews (4/6, 66%) lacked protocols or outcome specification, leading to low quality scores.

The strengths of our review include that all methods were guided by standards, including the Cochrane rapid review recommendations and AMSTAR 2 checklist. To enhance the ability of interventionists to use these findings, we focused on critical appraisal of quality and translatability through CASP checklists. We preregistered our protocol, and all accompanying data are supplied on an OSF website, which promotes reproducibility.

### Conclusions

As digital technology use is increasing, interventions to prevent problematic use and promote digital well-being will be of growing importance. This review demonstrates positive findings for effectiveness of prevention interventions for behavioral outcomes and describes factors that may contribute to translation and implementation. Digital well-being is a global need, and understanding contextual factors that influence healthy use as well as limitations of the current evidence is vital for informing future research. Future research would benefit from following appropriate reporting guidelines, reporting both the benefits and harms of interventions, and including greater detail on factors informing translation.

## Appendix

Multimedia Appendix 1 Supplementary materials.

Multimedia Appendix 2 PRISMA (Preferred Reporting Items for Systematic Reviews and Meta-Analyses) checklist.

## Abbreviations

AMSTAR: A Measurement Tool to Assess Systematic Reviews
CASP: Critical Appraisal Skills Programme
OSF: Open Science Framework
PICOS: population, intervention, comparator, outcome, and study design
RCT: randomized controlled trial
RQ: research question

## Data Availability

The datasets generated and analyzed during this study are available in the Open Science Framework repository [59].
