# Supplementary material for: Prevention and Health Promotion Interventions for Young People in the Context of Digital Well-Being: Rapid Systematic Review
Source: J Med Internet Res. 2024 Dec 18;26:e59968. doi: 10.2196/59968 (PMC11694046; doi:10.2196/59968)
Supplement: Multimedia Appendix 1 [file jmir_v26i1e59968_app1.docx]

Supplementary Materials

# PROSPERO Protocol (blinded)


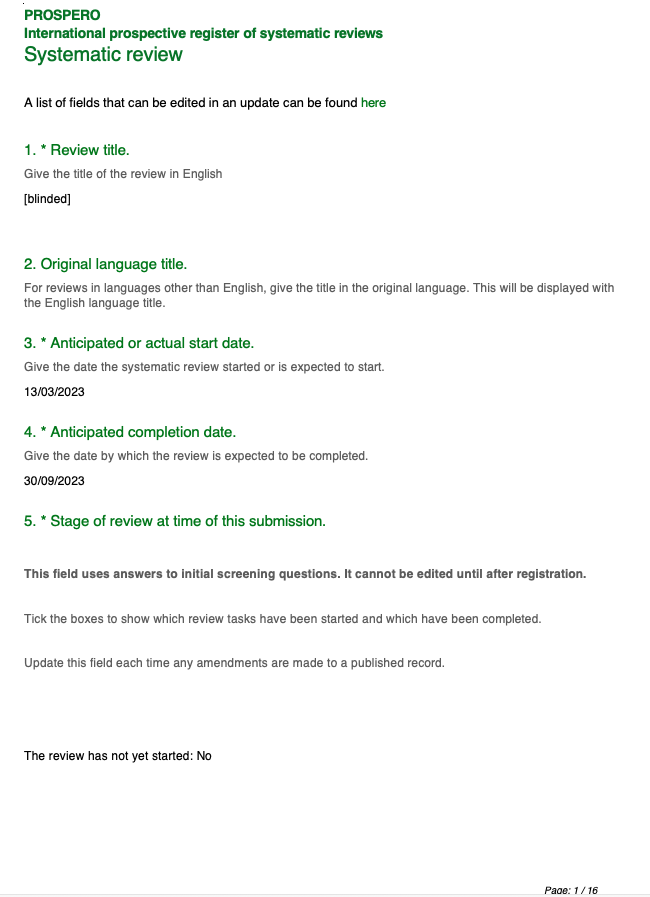


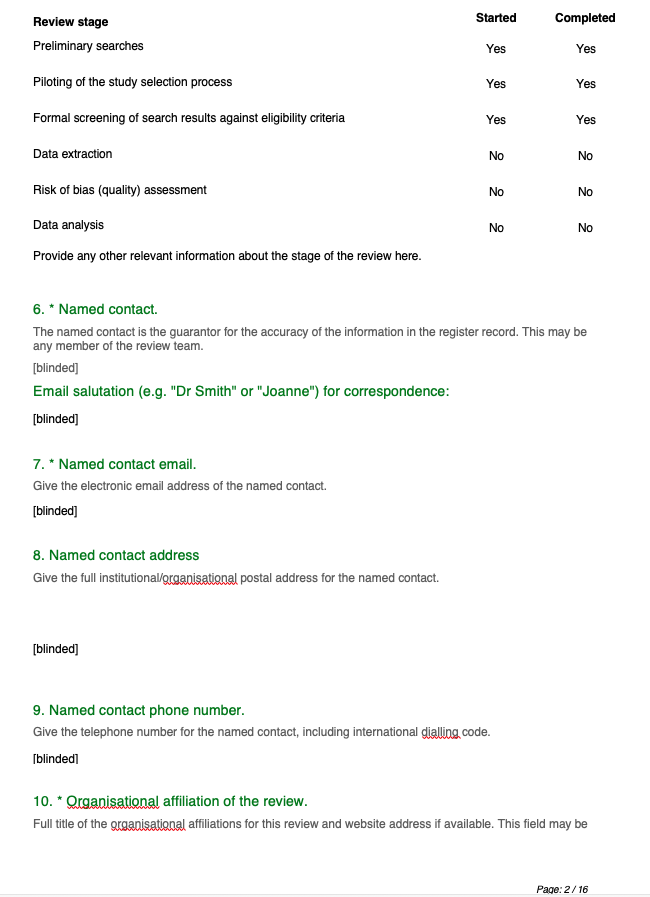


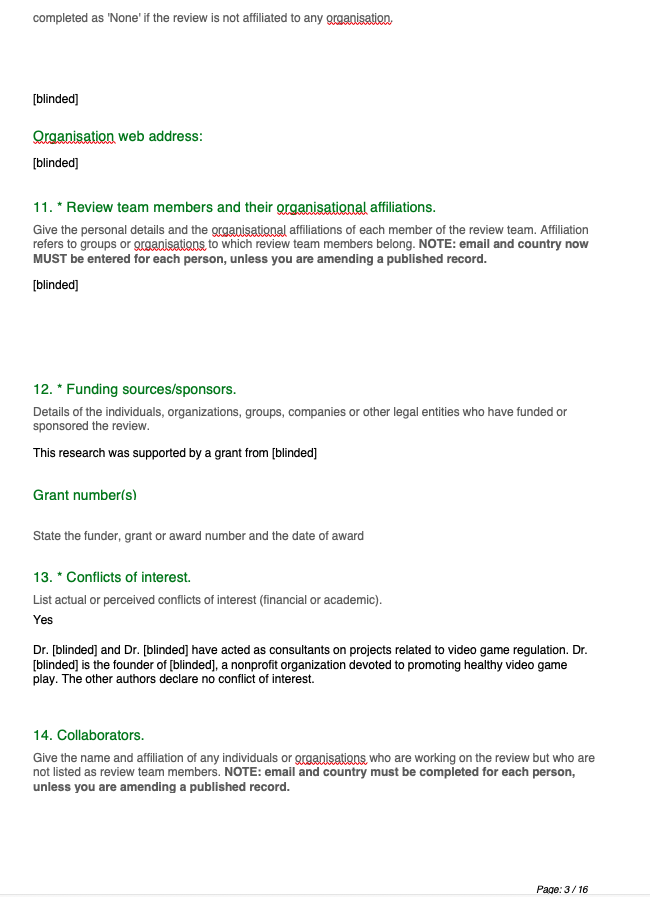


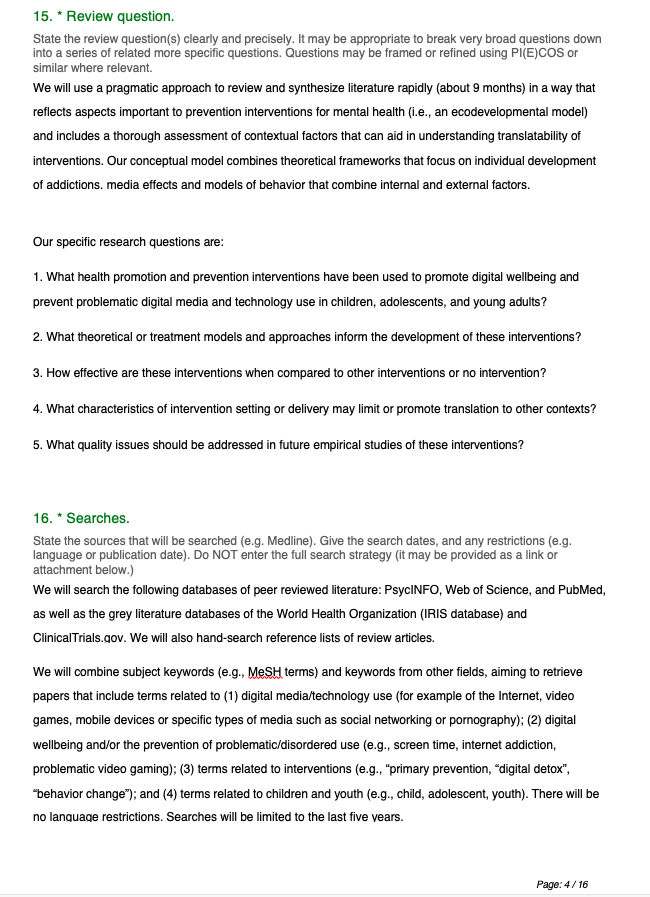


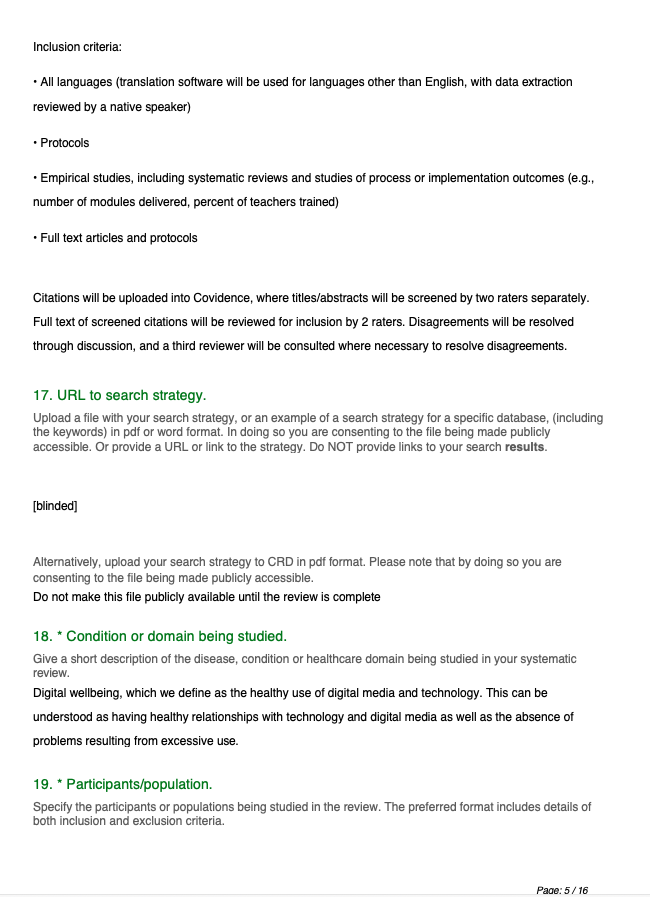

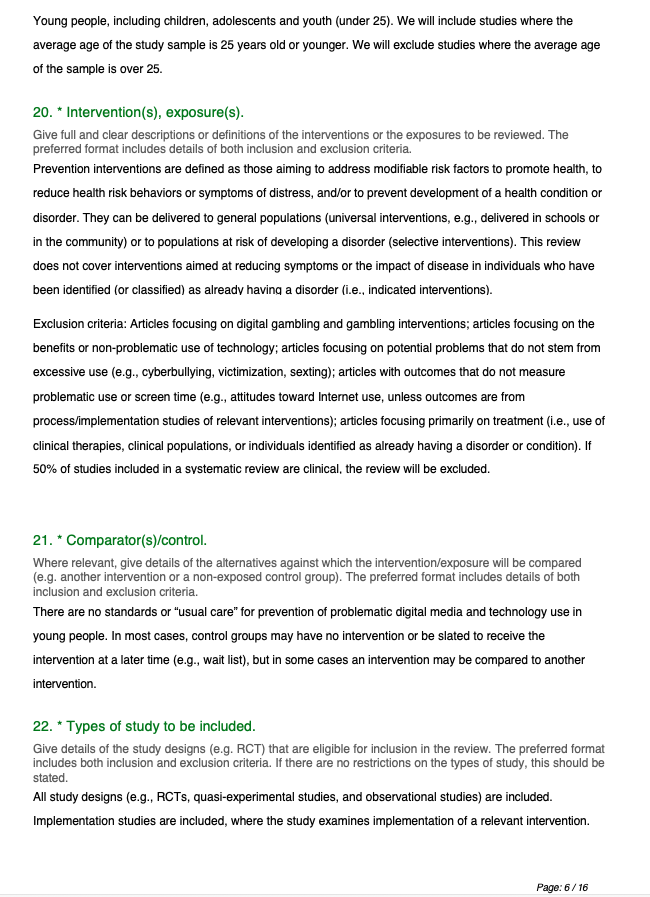


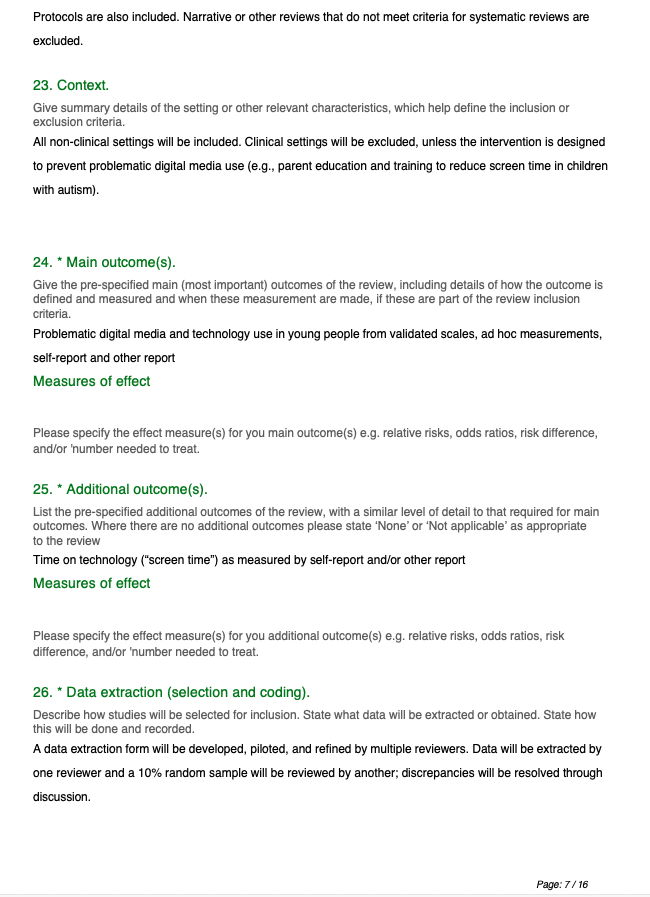


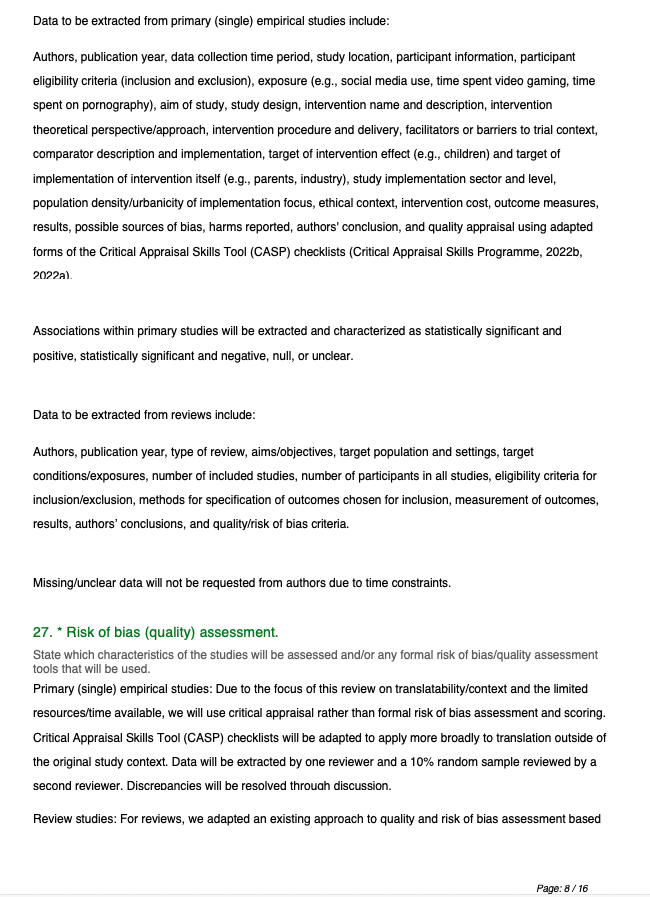


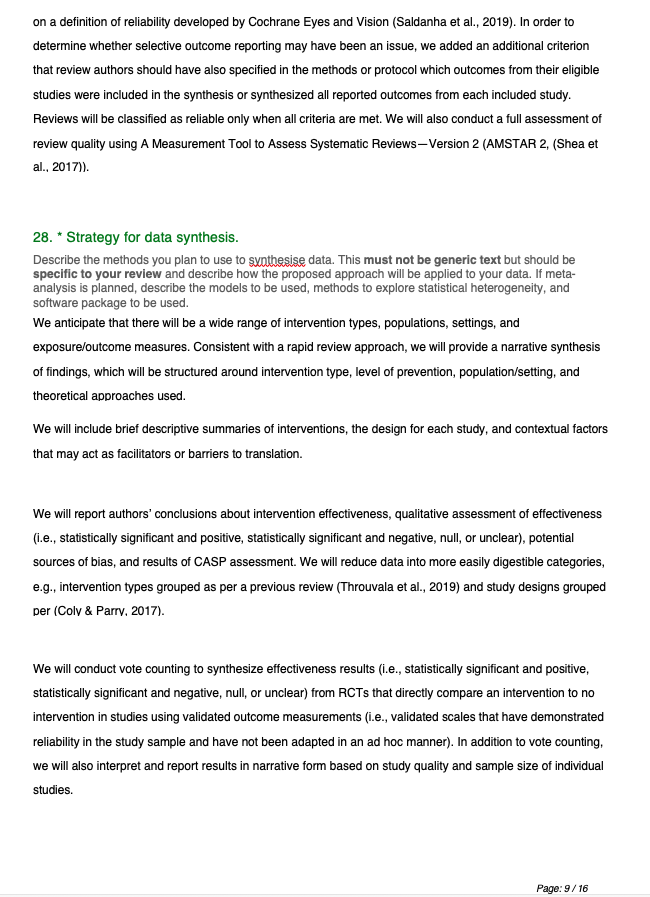


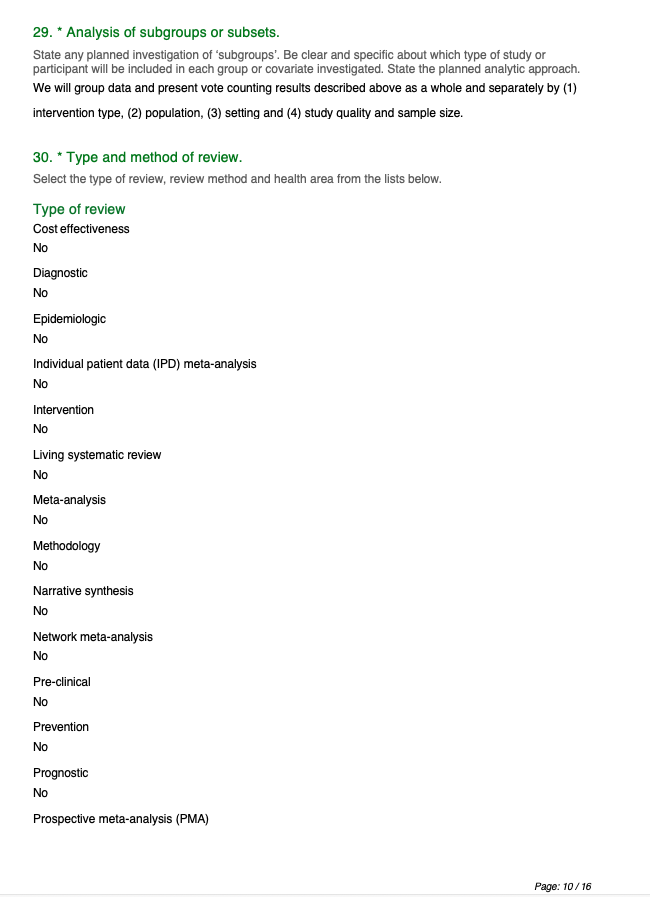


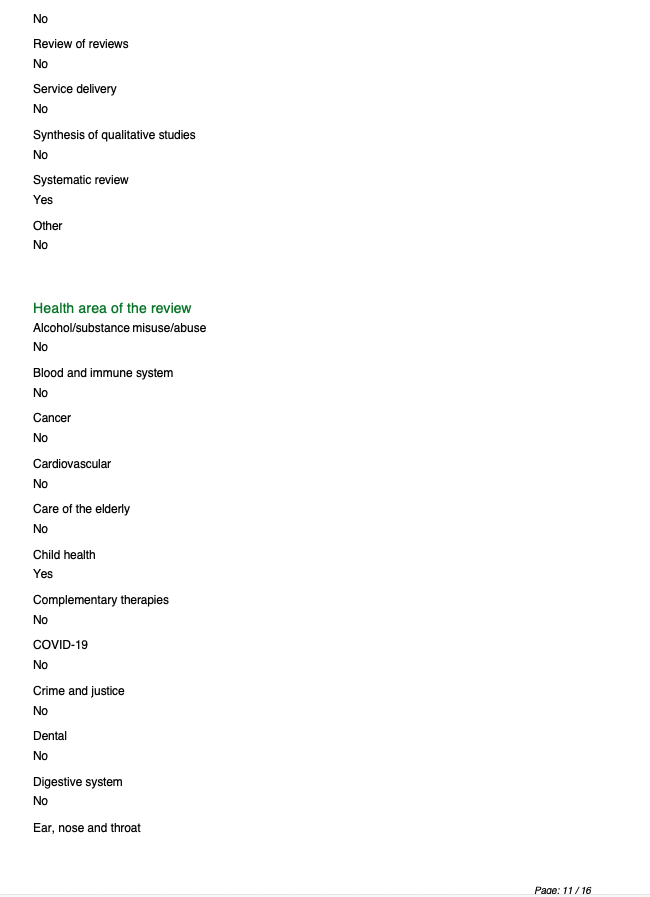


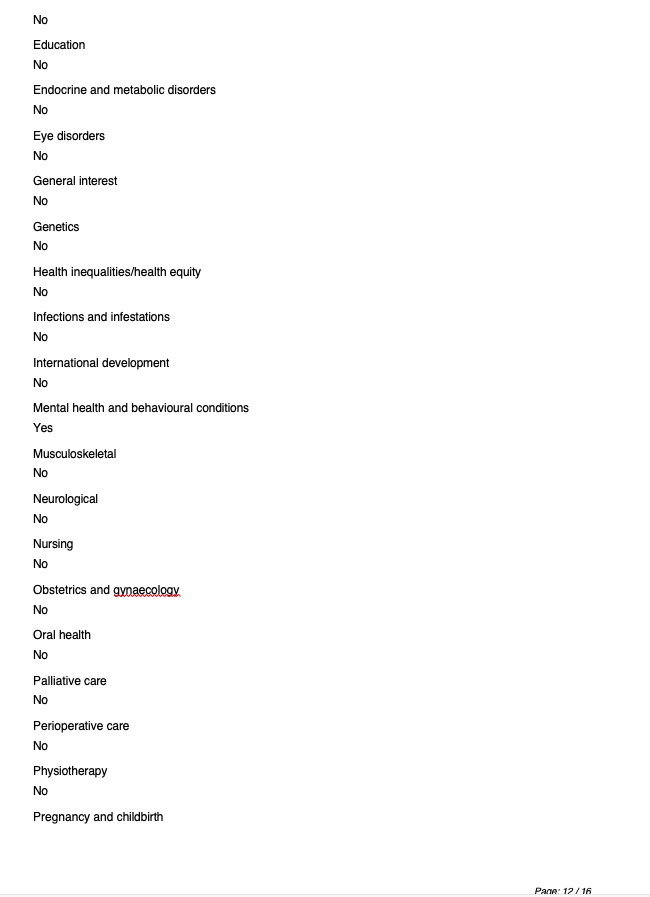


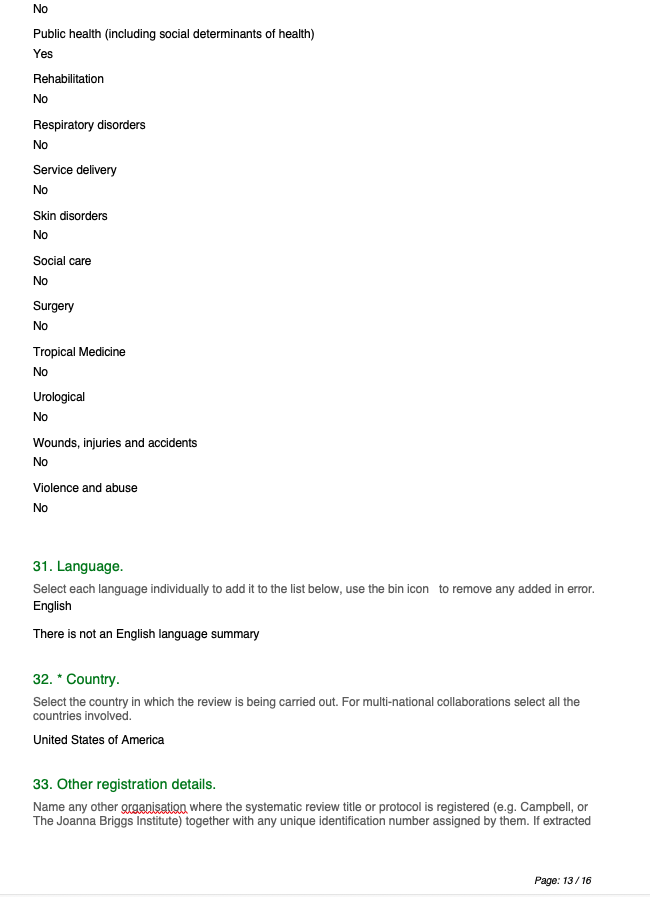


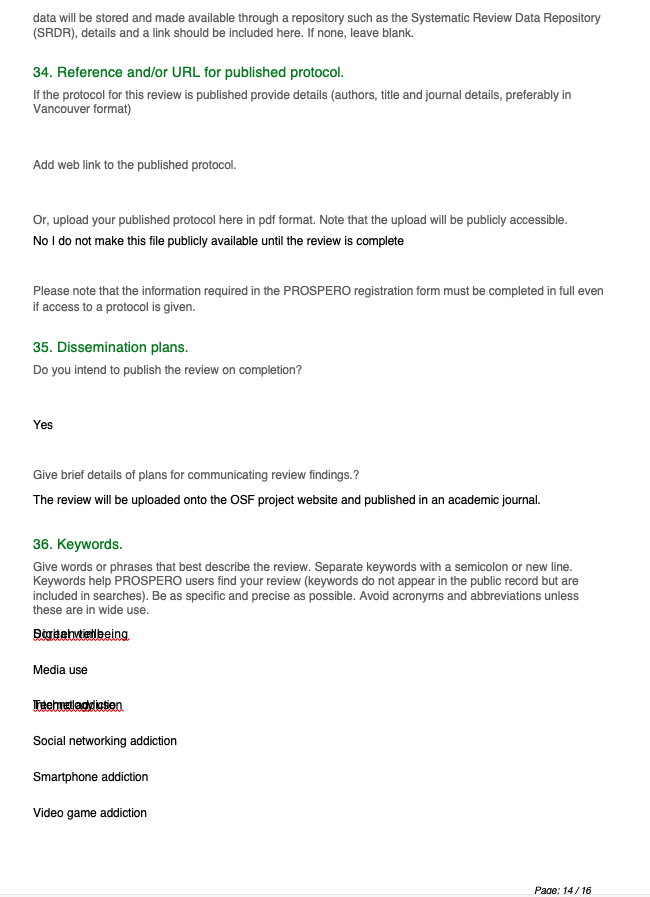


Note: The PDF generated by PROSPERO had some problems; here are the first keywords from a screen capture.


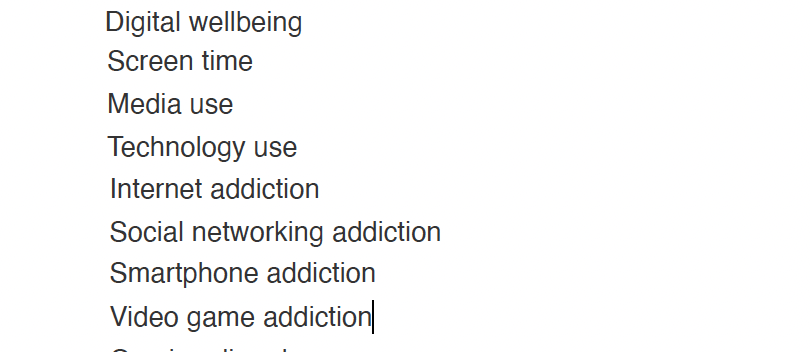


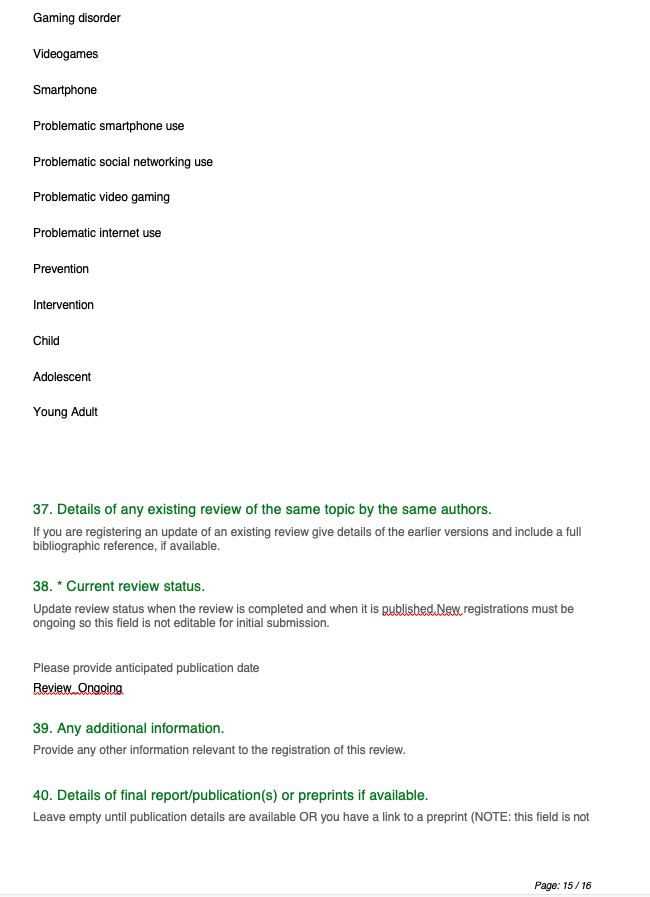


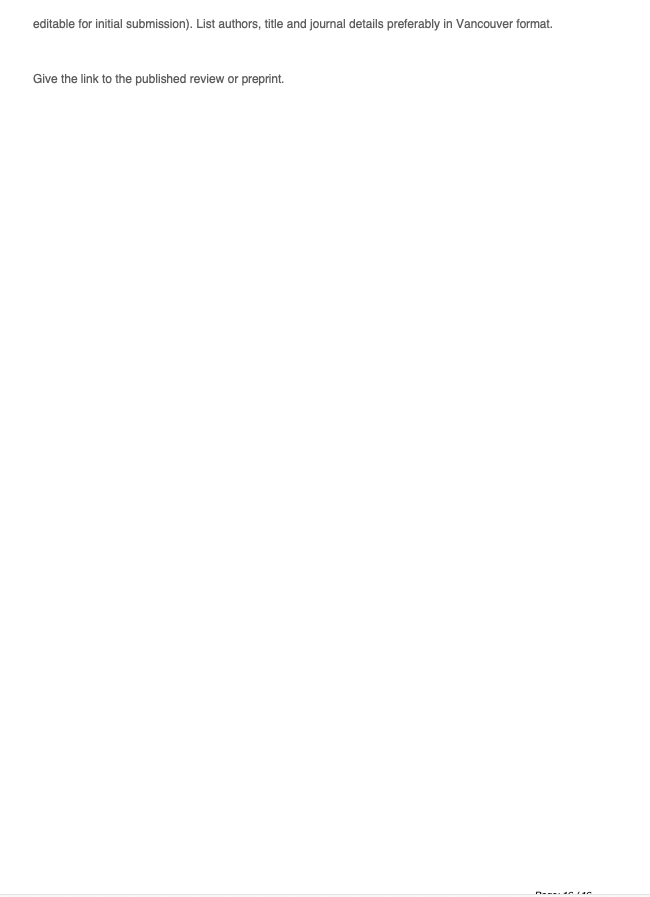


# PICOS and Search Strategies

Population: Children, adolescents and young adults (to age 25)

Interventions: Any prevention or health promotion intervention designed to address digital wellbeing

Comparisons: Any

Outcomes: Any measure of digital wellbeing, such as time spent using digital media or problematic use of digital media

Study designs/settings: Any study design and any setting

| **#** | **PsycINFO Query** | **Limiters/Expanders** | **Last Run Via** | **Results** |
| --- | --- | --- | --- | --- |
| S6 | (adolescen* OR "young adults" OR "young adult" OR teen* OR youth OR child*) AND (S1 AND S2 AND S3 AND S4) | Limiters - Publication Year: 2017-2023 Expanders - Apply related words; Apply equivalent subjects Search modes - Boolean/Phrase | Interface - EBSCOhost Research Databases Search Screen - Advanced Search Database - APA PsycInfo | 2,525 |
| S5 | (adolescen* OR "young adults" OR "young adult" OR teen* OR youth OR child*) AND (S1 AND S2 AND S3 AND S4) | Expanders - Apply related words; Apply equivalent subjects Search modes - Boolean/Phrase | Interface - EBSCOhost Research Databases Search Screen - Advanced Search Database - APA PsycInfo | 4,867 |
| S4 | adolescen* OR "young adults" OR "young adult" OR teen* OR youth OR child* | Expanders - Apply related words; Apply equivalent subjects Search modes -Boolean/Phrase | Interface - EBSCOhost Research Databases Search Screen - Advanced Search Database - APA PsycInfo | 1,363,976 |
| S3 | addict* OR problematic OR compulsive OR excessive OR disorder OR pathological OR heavy OR dependence OR "digital well-being" OR "digital balance" OR "digital wellbeing" | Expanders - Apply related words; Apply equivalent subjects  Search modes - Boolean/Phrase | Interface - EBSCOhost Research Databases Search Screen - Advanced "Search Database - APA PsycInfo" | 1,696,063 |
| S2 | interven* OR prevent* OR therap* OR treat* OR modif* OR detox OR parenting OR "behavior change" OR "behaviour change" OR program* | Expanders - Apply related words; Apply equivalent subjects Search modes - Boolean/Phrase | Interface - EBSCOhost Research Databases Search Screen - Advanced "Search Database - APA PsycInfo" | 2,346,016 |
| S1 | "Social media" OR Facebook OR Tik-Tok OR Tiktok OR Twitter or Instagram OR tumblr or Social Medium or facebook OR Pinterest OR youtube OR myspace OR wechat OR whatsapp OR reddit OR "social network" OR "social networking" ORSNS OR Weibo OR snapchat OR "online shopping" OR cyberporn OR "adult content" OR pornography OR videogame* OR "videogame" OR "video games" OR "computer game" OR "computer games" OR "online game" OR "online games" OR "Internet gaming" OR "commercial games" OR "mobile game" OR "mobile games" OR "screen time" | Expanders - Apply related words; Apply equivalent subjects Search modes -Boolean/Phrase | Interface - EBSCOhost  Research Databases  Search Screen - AdvancedSearch Database - APA PsycInfo | 77,765 |

# Table S1: Full Summary of Findings for Empirical, Non-Review Studies

| **First author name, year** | **Population & study design** | **Intervention** | **Relevant outcomes** |
| --- | --- | --- | --- |
| **Controlled studies** | |  |  |
| *Primary prevention* | |  |  |
| *Education* | |  |  |
| Apisitwasana 2018 | Cluster RCT of 310 4th and 5th grade students in two schools in Bangkok, Thailand and their parents. Measurements taken post intervention and at 3-month follow up. | Classroom activities based on self-regulation theory & participatory learning delivered by teacher 1 hr/week for 8 weeks. Educational material provided to parents, who provided weekly feedback. | Game addiction scores decreased from baseline and increased, but remained significantly lower than control. IG group showed significantly fewer days/week playing VG at follow up vs CG. No statistically significant differences in playing VG minutes per weekday or minutes per weekend day between CG and IG. |
| Bickham 2018 | Controlled before and after study of 412 6-8th grade students in the US, comparing one intervention school to a control school. Measurements taken before and after the intervention. | Take the Challenge: A school-based media education/reduction program based on social cognitive theory to motivate students to reduce screen time. Students complete a 6-week curriculum of classroom activities followed by a 10-day school-wide screen-free event. | Students receiving TtC had significantly less television viewing after school and on Saturdays, after-school video gaming, and Saturday Internet use post-intervention. Differences between groups in after school internet use and Saturday video game play were not significant. |
| Bonnaire 2019 | Controlled before and after study of 384 middle school students in randomly selected classrooms at five schools in Paris. IG had pre (T0), post (T1) and 3-month follow up (T2) assessments, but control groups did not have post-test (T1). | Single session prevention intervention designed to increase knowledge and skills: (1) awareness about screen time and its consequences and (2) reflection and skill building around life priorities and protective factors | Main analysis: At T2, there were more gamers with IGD in the control group vs. IG. At T2, IG had significantly fewer minutes/week and per weekend on Internet and video games compared to control after controlling for baseline differences.  Subgroup analysis: At T1, girls spent significantly more time on Internet (weekdays and weekends), and boys spent more time on video games (weekdays and weekend). At T2, girls spent more time on Internet during the week and boys spent more time playing video games during the week and weekend. At T1, the number of boys meeting IGD criteria increased, resulting in a significant difference, but was smaller again at T2, and there was no significant difference. |
| Esmaeili Rad 2018^a^ | RCT comparing intervention to control (no intervention) conducted with 200 college students in Urmia, Iran with pre- and post-assessments. | Two weeks' use of a mobile app based on reality therapy/choice theory and time management to measure and decrease social networking addiction. Students in the IG selected which apps would be restricted by the intervention. The app also suggested substitute activities based on initial input from users. | No direct comparison between IG and CG, but scores on the online social networking addiction scale decreased significantly post intervention in IG but not CG and there was a significant difference in time spent online in IG at post-test but not CG. |
| Gui 2023 | Cluster randomized trial of the impact of a teacher training course on 2,997 10th grade students in 171 classes in 18 schools in Northern Italy. Baseline measures taken in November 2017 and follow up in May 2018. | Digital Well-Being - Schools (DWB-S), a hybrid online and in-person training intervention to help teachers plan and implement media education activities for the promotion of healthy use of mobile media and the promotion of digital wellbeing. This prepares teachers to deliver four 3-hr Media Awareness Experience to 10th graders, including a smartphone time tracking app + modules based on the European Digital Competence Framework for Citizens. | Two subscales from two different measures of smartphone addiction were used to compare IG and CG. Based on ITT analysis, the IG had less problematic smartphone use in terms of "disturbance to adaptive functions" but not to withdrawal; there was no difference in social network use. There was a significant decrease in the withdrawal subscale in females compared to males post-intervention, but no differences in "disturbance to adaptive functions" or social media use, and the intervention did not show significant effects in males. |
| Hansen 2022 | Case-control study of students in participating schools in three federal states in Germany. Individuals in the intervention group were matched 1:1 on demographic characteristics with individuals in schools that had not received the intervention; a total of 834 children and adolescents participated. Measurements taken at baseline and at the end of the semester. | The "Net-Piloten" (Net Pilots) peer project for the prevention of social media addiction is designed to increase knowledge of negative consequences of social media use, promote self-regulation and attitude change, promote help-seeking when necessary. It is delivered to 6-7th graders in 2 90-minute workshops conducted by 8th-10th graders from the same school. | Video game play duration was lower in IG for school days and non-school days. Daily social media chatting on school-free days was lower in IG, but the difference in time chatting on school days was not statistically significant. CG spent significantly more time streaming on school days and non-school days. Problematic use was significantly lower in IG vs CG, and the proportion of students with low CIUS scores (<15) was greater in IG vs control. |
| Li 2019 | Cluster RCT comparing intervention schools (n=35) to control schools (n=35); 362 parents of Primary 4-6 grade gamers in Hong Kong, China. Post-test measurements collected 1 week (T2) and 3 months (T3) after intervention completion. | Game Over Intervention (GOI), based on ecological systems theory and self-determination theory, was delivered (unclear who delivered) as a single 4-hour intervention to parents. GOI is a universal parent-based intervention developed to empower parents with the knowledge, attitudes, and skills to cultivate positive parenting and family environments, which serve as protective factors against gaming disorder in children. Modules include Parental Monitoring, Parental Care, and Psychoeducation. Parents in the control group received a single 4-hour training on effective learning for children. | Gaming time and gaming disorder symptoms differed between IG and CG at baseline. The IG showed a significantly greater decrease post intervention than CG, but the groups were the same at 3-month follow up. Levels of gaming disorder (adapted observer version of Korean Internet Addiction Scale for Adolescents K-scale, modified to fit context of video gaming) did not differ significantly between CG and IG post-intervention or at follow up. |
| Marco 2017^a^ | Three-arm RCT (IG SIN, IG + additional impulse control techniques [IG+], wait list CG) of 1110 primary and secondary students in 9 public and 4 private schools in Valencia, Spain. IG compared to IG+ at post-test (T1) and follow up (T2), but no direct comparisons between IG/IG+ and CG. Measurements were taken at pre-test and again at follow up 2-5 months after intervention delivery. | PrevTec 3.1 for video games was delivered by trained psychologists/psychology students in the classroom in 50-minute sessions once a week for three weeks. The base intervention is designed to educate, raise awareness about, and promote healthy technology use and includes creating a schedule and many other activities. The IG+ intervention included additional impulse control techniques. | No significant change in CG from T0-T1. ANCOVA (controlling for age) showed group (IG v IG+) x time effects on days per week of play and video game dependence but not on minutes playing per weekday or minutes playing per weekend day. |
| Mumcu 2021^a^ | RCT of 110 schoolchildren in Turkey comparing intervention to no intervention with measurements taken pre- and post the 12-week intervention. | 1 hour of group recreational exercises were applied in school 3 days a week for 12 weeks to examine the effects of recreational activities on children's digital game addiction and peer relations. | In males, game addiction decreased significantly in IG and remained the same in CG. For females, there were no significant changes in either group. No direct comparisons were reported. |
| Ortega-Barón 2021 | Controlled pre-post study of 165 middle-schoolers in 3 regions of Spain, with measurements taken at baseline (December 2019) and post-intervention (March 2020). | The Safety.net program aims to prevent problematic Internet use, online grooming, nomophobia, and Internet gaming disorder in early adolescence. It is delivered by course tutors through 16 1-hour sessions in 4 modules and teaches digital skills, relational risks, dysfunctional risks, and change of attitudes and cognitions. | PIU increased in both groups but significantly more so in the control group. |
| Weaver 2022 | Controlled study of 65 students taking high school health classes in a single high school in the US who had an active social media account were recruited; 54 completed the intervention. Students in a different class were used as the control group. Measurements taken pre and post the 5-week intervention. | The intervention group underwent a five-week intervention called Mindful Connections, which involved learning wellness and stress-reduction techniques, as well as exploring the effects of social media on wellness. The intervention was delivered by a graduate student researcher in 50-minute groups with 8 -10 members per group, and the sessions occurred once a week. | Students in the IG reported significantly lower problematic social media use post-intervention compared to students in the control class. |
|  |  |  |  |
|  |  |  |  |
| *Community* | |  |  |
| Sanders 2018 | RCT comparing IG and waitlist control. Parents of children between the ages of 5 and 12 years old were recruited through locally placed and online advertisements. 32 families completed a second measurement and were included in the analysis. Effect sizes were estimated but no statistical tests of screen time were conducted. | The single-session, in-person intervention was delivered by a therapist and included a one-hour instructional period covering psychoeducation on children's media use, media management in the home, parenting skills, and parental controls, followed by a one-hour hands-on workshop where participants could receive individual assistance, ask questions, and learn how to enable parental controls on their devices using a resource guide. | No statistical tests were conducted in this pilot study, but parent estimates of overall screen time use over the past week (both weekdays and weekends) decreased more for IG v CG, but daily diary-reported screen time for specific types of media use each day for (collected for 7 days after baseline assessment and for seven days 6 weeks post-intervention) showed negligible decreases in each group. |
|  |  |  |  |
| *Online* | |  |  |
| Throuvala 2020 | RCT comparing intervention to no intervention in a convenience sample of university students in the UK who used mobile phones and social media. 143 were included in the per-protocol analysis with measurements taken pre- and post the 10-day intervention. | The intervention was designed to promote mindfulness, raise awareness of media and smartphone use, enhance self-regulation, and reduce distractions and time spent on smartphones and social media. IG was asked to use freely available mobile apps for daily self-monitoring of social media activity, mindfulness practices, and mood tracking for 10 days. The IG also received email reminders. | Hours per day of smartphone use and problematic social media use showed a statistically significant decrease in IG vs control, but there was no significant difference in hours per day of social media use. |
|  |  |  |  |
| *Secondary prevention* | |  |  |
| *Education* | |  |  |
| Affouneh 2021 | RCT with a convenience sample of 30 university students in West Bank, Palestine who "engaged in excessive Internet usage". Measurements taken at baseline and after the 8-week intervention. | Group training (CBT-based) to improve social skills and reduce internet addictive behaviors delivered by a psychologist during two 90-minute sessions/week for 8 weeks | IG showed significantly less internet addiction vs CG post-test |
| Lindenberg 2022 | Multicenter cluster RCT comparing IG and assessment-only control group of high school students in Germany. 5549 participants were screened; 422 had elevated symptoms of GD and unspecified IUD and were randomized. Measures assessed at baseline, 1 month, 4-month, and 12-month follow-ups. | PROTECT is a theory-driven, manualized, cognitive behavioral therapy (CBT)–based preventive group intervention delivered in schools that consists of four 90-minute sessions and is delivered by two trained psychologists. | Symptom severity of combined gaming disorder & unspecified internet use disorder (measured by a single modified scale) decreased significantly in the IG vs CG. Harms were measured and an equal number of participants developed unspecified Internet use disorder (as assessed by 5+ DSM criteria on diagnostic interview) in each group. Differences in incidence rates of GD between IG and CG were not significant. |
| Tang C 2021 | Cluster RCT of two middle schools (Intervention and control schools) in Zizhong County, a rural area of the Sichuan province, China. 775 students with excessive use of internet constituted the study sample. Measures were collected at baseline and after each wave of intervention (every 6 months) for a total of 5 assessments. | The intervention was primarily provided by teachers in health education courses and was split into 4 parts lasting roughly 45 minutes each, provided roughly every six months. The third intervention included 1:1 guidance, while the final phase of the intervention included sports equipment that could be used during class | Between baseline and the fifth survey, the proportion of excessive users decreased from 100% (inclusion criteria) to 70% at the last survey, but no statistical test was reported. IG and CG were not compared in terms of problematic use/behaviors, but within the IG, the proportion of those who used the internet for 4+ hours/day on weekends decreased significantly from baseline to follow up, as well as the proportion of those gaming 2+ hours a day on weekdays. No improvement was observed in the proportion spending 2+ hours/day online on weekdays or on the proportion of those being online overnight in the past month. |
|  |  |  |  |
| *Online* |  |  |  |
| Brailovskaia 2022 | RCT with three arms conducted on young adult smartphone users (at least 75 min/day). Participants recruited from community advertisements and through social media in Germany, n=619 completed and were analyzed. Baseline measures (T0) were compared with post-intervention (T1), 1 month later (T2) and 4 months post intervention (T3). | The abstinence group waived their daily smartphone use for 7 days, while participants of the reduction group reduced their daily smartphone use by 1 hr based on initial reports of average daily use. IG participants (but not CG) also completed a compliance diary each day. | The abstinence group showed significantly less daily smartphone use than control at T3 and T4 post-intervention, but there were no significant differences between the reduction and abstinence groups or the reduction and control groups at any measurement time point. Compared to the control group, the reduction group showed lower problematic smartphone use (PSMU) at T3 and T4, but there were no differences at T2. There were also no significant differences between reduction and abstinence groups or abstinence and control groups in PSMU at any measurement time point. |
| Ko 2021^c^ | Controlled pre-post study of 16 young adults recruited online who perceived themselves as social media addicts and wanted to decrease their social media usage, with measurements taken at baseline and after the 2-week intervention. | A newly developed mobile app, Social Media Addiction Coach, asked for users' preferred exercise and social activities and social media use times, suggested alternative activities during those times every day, and sent notifications to remind users to engage in the alternative activity. Users uploaded proof that they did the alternate activities and sent screenshots of time use daily. IG-1 was asked to substitute social activities for social media use during the first week, then to substitute exercise (e.g., yoga) for social media use the second week; the order was inverted for IG-2. The control group only reported daily screen time. | There were no significant differences in social media use between either IG and CG or between the two intervention groups. |
| Thai 2023 | RCT of 260 (220 completed) college students who regularly used social media (>45 min/day) in Canada who had symptoms of depression or anxiety were randomized to intervention or control. Measurements occurred at baseline and post-intervention. | IG was asked to limit social media use to 1 hour/day for 3 weeks and was sent daily reminder emails. All participants were instructed to send daily screenshots of their smartphone's screen time tracker for the duration of the study. | Compared to CG, IG showed significantly less smartphone use at the end of the intervention. |
|  |  |  |  |
| **Pre-post studies without control groups** | | |  |
| *Primary prevention* | |  |  |
| *Education* | |  |  |
| Chau 2019 | Before and after study of 248 primary school students in Hong Kong, China. Baseline measures (T0) were compared with post-intervention (T1) and 1 month later (T2). | Wise IT-use program, a hybrid online and in-school intervention built on gamification and flow theory. It takes place over 3 months that enhances students’ awareness of IGD and risky online behaviors and educates about how to prevent them. | Statistically significant difference in the distribution of normal, at-risk and high-risk gamers between T1 and T2; the proportion of “at-risk gamers” declined from 6% to 2%, whereas the proportion of high-risk gamers was the same. Levels of IGD symptoms decreased from T1 to T2 by two points; this was statistically significant. |
| Ke 2018a | Pre-post study of teenaged students from government secondary schools in Malaysia selected by school counselors on the basis of having potential PIU and being motivated to change their behavior. 45 participants completed the program. Measures were completed at baseline (T1), post-intervention (T2), and 1-month follow up (T3). | The Psychological Intervention Program-Internet Use for Youth (PIP-IU-Y) is a CBT-based program designed for adolescents that aims to prevent development of PIU by promoting healthy coping and improving face-to-face social interactions. Eight 90-minute group sessions are delivered by school counselors. | Participants showed statistically significant decreases in PIU between time points (T1-T2, T2-T3, and T1-T3). |
| Ke 2018b | Pre-post study of teenaged students from government secondary schools in Malaysia selected by school counselors on the basis of having potential PIU and being motivated to change their behavior. 157 participants completed the program. Measures were completed at baseline (T1), post-intervention (T2), and 1-month follow up (T3). | The Psychological Intervention Program-Internet Use for Youth (PIP-IU-Y) is a CBT-based program designed for adolescents that aims to prevent development of PIU by promoting healthy coping and improving face-to-face social interactions. Eight 90-minute group sessions are delivered by school counselors. | Participants showed statistically significant decreases in PIU between time points (T1-T2, T2-T3, and T1-T3). |
| Kent 2021 | Pre-post study using a convenience sample of 10 undergraduate students in the United Kingdom. Participants were required to be undergraduate students who had problematic Internet use as determined by IAT (all participants had mild to moderate problematic use rather than clinically diagnosable levels), were contemplating change, and used Android smart phones. Measurements were taken pre- and post-intervention (one month). | An intervention based on the stages of change model was delivered via a smartphone application over the course of one month. Participants were invited to set goals around smartphone use and other behaviors for 10 days, then had one week each of personalized feedback, mindfulness, then behavioral messages. Participants received a message each day for seven consecutive days within each of these phases. | Eight of 10 participants showed improvement in problematic mobile phone use; 7/10 showed "reliable improvement". One showed a "reliable deterioration". Nine out of 10 students showed improvement in IAT, but only 3/10 showed "reliable improvement". (Screen time was reported for each individual graphically but this was not extracted.) |
|  |  |  |  |
| *Online* |  |  |  |
| King 2017 | 27 young adult MMO players were recruited online; 24 completed the intervention and were analyzed at one week and four weeks after. | Participants were asked to refrain from gaming over a single weekend (84 hours, Friday 12:00am to Monday 12:00 pm) and received daily surveys. | Although there was not a significant trend in all participants to have fewer gaming hours between baseline and 28 days, those with IGD had a significantly greater decline in gaming activity. IGD symptoms declined significantly over time, and the decline was greater in those with IGD. Most of those with IGD experienced reliable improvement between baseline and 28 days, but fewer showed reliable improvement in gaming time. A smaller proportion of the non-IGD group showed reliable improvement in IGD symptoms, however; one participants experienced a clinically significant deterioration. |
|  |  |  |  |
| *Secondary prevention* | | |  |
| *Community* | |  |  |
| Männikkö 2022 | Pre-post study of 37 young adults recruited from five cities in Finland who self-reported excessive gaming and were willing to get support in monitoring their behavior. Data were collected at baseline (T0), post-test (T1), and 6-month follow-up (T2). | The Limitless Gaming Bootcamp program provides group interventions for intense gamers who seek to reduce the effects of gaming on their everyday lives and consists of instructor-guided group discussions, skill training, and/or behavior change assignments and common activities held in 10 weekly sessions of 3 hours each over 3 months. Sessions included a weekly theme, social gaming, and wrap-up/reflection. These were delivered by an experienced healthcare professional in collaboration with a "peer coach", a young adult volunteer with a personal history of problematic gaming who had completed extensive training. | Participants demonstrated a slight reduction in the severity of problematic gaming over assessment periods, which was supported by a significant decreasing trend between pre- and post-intervention. There were no significant differences in gaming time, internet use, or TV viewing time. |
|  |  |  |  |
| *Other* | |  |  |
| Heffler 2022 | Pre-post study of 9 preschool-aged children diagnosed with autism who viewed screens for at least two hours a day. Parents were recruited through community healthcare providers in Pennsylvania, United States. Measurements taken at baseline and post-intervention (6 months). | Parents viewed a 40-minute educational video, then attended weekly, one-hour in-home support visits from a trained therapist for six months to receive tailored instruction on how to replace screen time with social engagement time. | Parents' reports of children's screen time decreased from an average of almost 6 hours a day to less than 5 minutes a day at the end of 6 months (no statistical test as this was not a primary outcome). No adverse effects of keeping screens off were reported, and parents felt motivated to keep screens off by observing early changes in their children. |
|  |  |  |  |
| **Observational studies & protocols** | | |  |
| *Primary prevention* | |  |  |
| *Education* | |  |  |
| Barker 2021 | Protocol only; program would target preadolescent students in the US who play online games | Mindfulness & DBT group psychoeducation and skills building to prevent problematic internet gaming. Weekly 2-hour sessions for 8 weeks delivered by a trained facilitator and co-facilitator. | N/A; protocol only |
| Hansen 2021 | A cross-sectional survey was conducted to evaluate implementation of the Net Piloten (Net Pilots) program across Germany. All 542 "multipliers" who had been trained across Germany to deliver the program were contacted; 210 completed the survey. No statistical testing was reported. | This evaluation project evaluated implementation of training and provision of the "Net-Piloten" (Net Pilots) peer project for the prevention of social media addiction (Hansen 2022 in this review). "Multipliers" were trained to train peer intervention providers (students). The intervention is delivered to 6-7th graders in 2 90-minute workshops conducted by 8th-10th graders from the same school. | This process evaluation found that implementation was low; only 26% of trained multipliers had trained others and 38% were able to train "net pilots". Many multipliers found the 4-day training session for students too time-consuming and found that their organizations had too little knowledge about the project, or that there were not enough staff to implement it. They also felt that content should be constantly updated to match changing trends in media use behavior. |
| Neverkovich 2017^b^ | Controlled trial of 657 high school, college, and medical school students in Moscow and Irkutsk, Russia. No time periods were reported. | The intervention consisted of psychological and educational support programs providing motivational, cognitive, practice-oriented, and reflective units. These occurred through 6 "events" for various groups such as social pedagogues, psychologists, teachers, teenagers, and parents including literature review, interviews and slide show, survey, parent meeting, discussions about prevention programs, and rules for avoiding addiction to social networks. | Descriptive statistics show pre- to post-intervention changes in proportions of participants identified as having various levels of internet addiction and social network addiction in both intervention and control groups, but no statistical tests are reported. |
| Tang A 2021 | Protocol for a cluster-randomized RCT with a proposed sample of 240 level 5 primary school students in Hong Kong, China. | The intervention is a 12-week mindfulness-based cognitive program (MBCP) aimed at improving adolescents' resilience, changing their smartphone behavior, and reducing smartphone addiction symptoms. It comprises 90-minute supervised practice at school and daily home practice which will be logged daily and checked by the trainer. The intervention group receives the MBCP, while the control group will receive existing counseling services for students with emotional/behavioral problems as treatment as usual. The effects of the intervention will be evaluated at five measurement points over a three-month period, including a three-month follow-up. | N/A; protocol only |
|  |  |  |  |
| *Community* | |  |  |
| Krossbakken 2018 | A post-test only experimental study of 1762 (1657 analyzed) guardians of young children (8-12) in Norway, recruited from a random sample of 5,864. 831 were mailed the intervention brochure and both groups assessed four months later. | The intervention consisted of a single, mailed, brief parental guide on “how to regulate video game behavior in children” based on clinical recommendations and experience, research on factors identified as useful for preventing video game problems, and feedback from parents. | There were no significant differences between groups at post test on gaming time on weekdays or weekends and video game problems (IGD criteria), although the subgroup of parents who reported reading and following the guide also reported more gaming problems than the other subgroups when compared to control. |
| Sela 2021 | Protocol only; 297 parents and their children in Israel proposed as participants, with proposed collection pre, post (3 weeks) and at 8-week follow up | The protocol describes three intervention groups: (1) training and assistance in setting parental controls (TPM); (2) training in Parental Vigilant Care (PVC), a parenting approach where parents tailor their level of active involvement in children's lives in a flexible manner, specific to the risks presented by their children (3) combining both group parental training and installation of filtering devices (PVC +TPM). | N/A, protocol only |
|  |  |  |  |
| *Online* | |  |  |
| Hayes 2022 | Protocol only, the study was terminated due to inability to recruit and retain sufficient participants | A 6 session, online, group-based, psychological course targeting problematic smart phone use | N/A, study was terminated |
| Schmuck 2020 | Cross-sectional online survey of 500 young adults in Austria who used a smartphone | Self-reported existing use of one of several digital detox apps for smartphone such as iOS Screen time, Android Digital Well-Being, Moment, Forest, Quality Time, Detox, Space, OffTime, RealizeD or similar | Use of social networking apps was associated with high problematic smartphone use (PSMU), as was use of digital detox apps. However, inclusion of an interaction term (digital detox app use x social networking app use) showed a qualitative difference: as social networking app use increased, PSMU decreased for digital detox app users |
|  |  |  |  |
| *Other* | |  |  |
| Choi 2018 | Natural experiment using results from a yearly panel survey of a representative sample of 243,957 middle and high schoolers in Korea who participated in the Korea Youth Risk Behavior Web-based Survey from 2005 to 2015 | Article 26 of the Juvenile Protection Act (shutdown policy): “Internet games should not be offered to those under the age of 16 from 12:00 am to 6:00 am.” In other words, the policy restricts adolescents aged 15 or below from playing online games after midnight (12:00–6:00 am). | After the policy was implemented, a reduction in problematic use (>300 min/day, called "addiction" in the study) was seen only in 2015 compared to 2011--no reduction seen from 2012-2014. Weekly internet use among 15-year-olds decreased significantly from 2012-2013, but the decrease in 2014 was not significant, and there was an increase in 2015. Internet use on weekdays and on weekends followed a similar trend. |
|  | | | |

*Note: ^a^Excluded from vote counting because lacked direct comparison between IG and CG; ^b^Excluded from vote counting due to unclear study design and analysis. ^c^Excluded from vote counting for unclear analysis reporting (direction of effect unclear). GD=Internet gaming disorder; IA=Internet addiction; IG=Intervention group; CG=Control group*
